# Antagonism Between Gut *Ruminococcus gnavus* and *Akkermansia muciniphila* Modulates the Progression of Chronic Hepatitis B

**DOI:** 10.1016/j.jcmgh.2023.12.003

**Published:** 2023-12-12

**Authors:** Huey-Huey Chua, Ya-Hui Chen, Li-Ling Wu, Hung-Chih Yang, Chia-Ray Lin, Huey-Ling Chen, Jia-Feng Wu, Mei-Hwei Chang, Pei-Jer Chen, Yen-Hsuan Ni

**Affiliations:** 1 Department of Pediatrics, National Taiwan University College of Medicine and Children’s Hospital, Taipei, Taiwan; 2 Department and Institute of Physiology, National Yang-Ming Chiao-Tung University College of Medicine, Taipei, Taiwan; 3 Department of Internal Medicine, Graduate Institute of Clinical Medicine, National Taiwan University College of Medicine, Taipei, Taiwan; 4 Graduate Institute of Microbiology, National Taiwan University College of Medicine, Taipei, Taiwan; 5 Graduate Institute of Medical Education and Bioethics, National Taiwan University College of Medicine, Taipei, Taiwan; 6 Center of Genomic and Precision Medicine, National Taiwan University College of Medicine, Taipei, Taiwan; 7 Hepatitis Research Center, National Taiwan University Hospital, Taipei, Taiwan; 8 Medical Microbiota Center, National Taiwan University College of Medicine, Taipei, Taiwan

**Keywords:** Bile Salt Hydrolase, Cholestyramine, Cholic Acid, Immune Active

## Abstract

**Background & Aims:**

A long immune-tolerant (IT) phase lasting for decades and delayed HBeAg seroconversion (HBe-SC) in patients with chronic hepatitis B (CHB) increase the risk of liver diseases. Early entry into the immune-active (IA) phase and HBe-SC confers a favorable clinical outcome with an unknown mechanism. We aimed to identify factor(s) triggering IA entry and HBe-SC in the natural history of CHB.

**Methods:**

To study the relevance of gut microbiota evolution in the risk of CHB activity, fecal samples were collected from CHB patients (n = 102) in different disease phases. A hepatitis B virus (HBV)-hydrodynamic injection (HDI) mouse model was therefore established in several mouse strains and germ-free mice, and multiplatform metabolomic and bacteriologic assays were performed.

**Results:**

*Ruminococcus gnavus* was the most abundant species in CHB patients in the IT phase, whereas *Akkermansia muciniphila* was predominantly enriched in IA patients and associated with alanine aminotransferase flares, HBeAg loss, and early HBe-SC. HBV-HDI mouse models recapitulated this human finding. Increased cholesterol-to-bile acids (BAs) metabolism was found in IT patients because *R gnavus* encodes bile salt hydrolase to deconjugate primary BAs and augment BAs total pool for facilitating HBV persistence and prolonging the IT course. *A muciniphila* counteracted this activity through the direct removal of cholesterol. The secretome metabolites of *A muciniphila*, which contained small molecules structurally similar to apigenin, lovastatin, ribavirin, etc., inhibited the growth and the function of *R gnavus* to allow HBV elimination.

**Conclusions:**

*R gnavus* and *A muciniphila* play opposite roles in HBV infection. *A muciniphila* metabolites, which benefit the elimination of HBV, may contribute to future anti-HBV strategies.

## Graphical abstract

**Figure:**
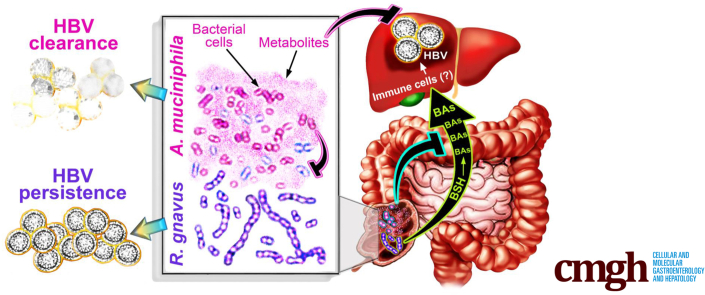

**Summary**

We deciphered the mechanism of the gut microbiota in controlling the development of chronic hepatitis B. *Ruminococcus gnavus* uses bile acids as mediators to promote immune tolerance, whereas *Akkermansia muciniphila* restrains this effect by stimulating immune activation to achieve early HBeAg seroconversion.

Chronic hepatitis B (CHB) poses a heavy disease burden worldwide.^1^ In the natural history of CHB, patients experience alanine aminotransferase (ALT) flares with liver inflammation resulting from damage to hepatitis B virus (HBV)-harboring hepatocytes that are attacked by the host immune response.^2^ If the immune reaction is appropriate, the virus may be eliminated, and the patients undergo HBeAg seroconversion (HBe-SC); if the immune reaction is prolonged and vigorous, the patients may suffer from serious complications such as liver failure, progressive fibrosis, cirrhosis, and hepatocellular carcinoma (HCC); if the immune reaction is not sufficient, it is usually clinically asymptomatic and HBV infection persists.^2^

Many CHB patients acquire infection through the mother-to-infant route, leading to a symptom-free and normal ALT in childhood and even young adulthood because they tolerate the viral antigens as part of their bodies.^3^ Later, some CHB patients experience elevated ALT levels and clear the virus because their immune system is fully activated to break the tolerance.^3^ Thus, one of the intriguing questions regarding CHB treatment is to uncover which trigger factors initiate this immune activation.

The intestine is the largest organ harboring immunologic cells, and the resident microbiota are the key players of our immune system.^4^ Previously, we showed that the gut microbiota contribute to the age-dependence of HBV immunity in an HBV-hydrodynamic injection (HBV-HDI) mouse model.^5^ Young C3H/HeN mice, which have not established a gut microbiota balance, demonstrate a tolerance phenotype such as prolonged hepatitis B s antigen (HBsAg) persistence, high HBV DNA titer, and impaired anti-HBs production.^5^ Sterilization of the gut microbiota using antibiotics prevents adult mice from rapidly clearing HBV and restores the tolerance phenotype,^5^ implying that the gut microbiota may transmit signals to break liver tolerance and evoke rapid HBV clearance. We hypothesized that the wax and wane of gut microbiota signatures may determine the progression of CHB. We aimed to delineate what the pivotal bacteria are and how they manipulate the progression of CHB. Once the responsible bacteria and their mechanisms are unraveled, it will certainly inspire some novel CHB therapies.

## Results

### Gut Microbiota Regulate the Natural Progression of CHB

**Figure 1:**
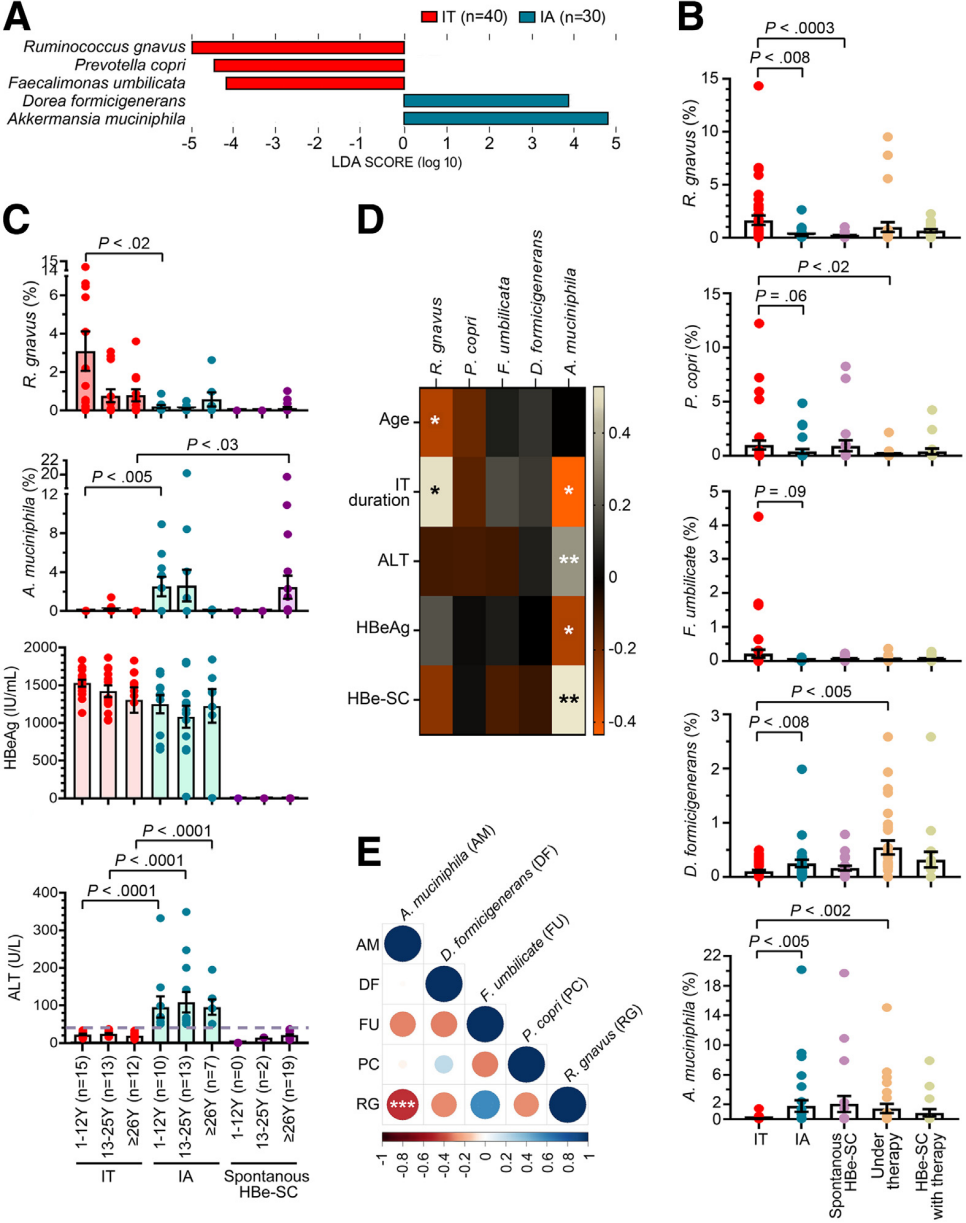
**Association between gut microbiota and CHB progression.** (*A*) Histogram of LDA score of gut microbiota between IT and IA patients. Only features with LDA score >3.5 and *P* <.05 are shown. (*B*) Relative amounts (%) of indicated bacteria of each group are presented as aligned *dot plots with bar graphs* of means ± standard error of the mean (SEM). Mann-Whitney test was used to compare groups including IT (n = 40), IA (n = 30), spontaneous HBe-SC (n = 21), under therapy (n = 28), and HBe-SC with therapy (n = 18). (*C*) Fecal levels of *R gnavus* and *A muciniphila* and serum levels of HBeAg and ALT in patients with different ages and disease courses were compared. Results are given as *dot plots with bar graphs* of means ± SEM. Differences between groups were evaluated by Mann-Whitney test. (*D*) Heat map shows the correlation between bacterial levels and clinical parameters as indicated. Pearson R correlation analysis was applied to find the significant features. The bacterial levels of IT, IA, and spontaneous HBe-SC groups were compared for their correlation with age and serum levels of ALT and HBeAg. The association between bacterial levels and IT duration was calculated using data of 1–12-year age group. Bacterial data sets of IA group were further estimated for correlation with the occurrence of HBe-SC. ∗*P* < .05, ∗∗*P* < .005. A *color-coded correlation scale* is provided on the right of the plot. (*E*) Matrix of Spearman rank correlation coefficient from comparisons of indicated bacteria detected in all samples (n = 137) is shown. The correlogram was generated using corrplot package in R. The Spearman correlation was evaluated among the indicated bacterial and displayed significance (∗∗∗*P* < .0007) between *R gnavus* (RG) and *A muciniphila* (AM).

To explore the impact of the gut microbiota on CHB, fecal samples (n = 137) were collected from 102 patients who were in different disease phases of CHB including immune tolerant (IT, n = 40), immune active (IA, n = 30), spontaneous HBe-SC (n = 21), and HBe-SC with antiviral therapy (n = 18). The other 28 stool samples were obtained from CHB patients receiving 3–12 months of anti-HBV therapy. Profiling the gut microbiota was achieved by 16S-rRNA next-generation sequencing (NGS). The differences in bacterial compositions were identified by linear discriminant analysis (LDA) effect size (LEfSe). With minimum LDA scores of 3.5 and statistical significance (*P* < .05, by Kruskal–Wallis test), *Ruminococcus gnavus* and *Akkermansia muciniphila* were revealed to be the most prominent bacterial species discriminating the IT and IA groups, respectively (Figure 1*A*). The significant differences in microbial taxa between groups were further validated by the Mann-Whitney test. Only *R gnavus*, but not *Prevotella copri* and *Faecalimonas umbilicata*, was particularly enriched in IT cases, with significantly higher abundances than that in the IA (*P* < .008) and spontaneous HBe-SC groups (*P* < .0003, Figure 1*B*). *Dorea formicigenerans* was highly colonized in the IA group compared with IT patients (*P* < .008), although less significantly than that of *A muciniphila* (*P* < .005, Figure 1*B*). Down-regulation of *P copri* (*P* < .02) and up-regulation of *D formicigenerans* (*P* < .005) and *A muciniphila* (*P* < .002) were observed upon anti-HBV treatment compared with IT patients (Figure 1*B*), indicating the microbial community changed in response to drug therapy.

Because the gut microbiota–mediated immune clearance of HBV infection is age-dependent,^5^ we next analyzed the microbial distribution in accordance with age. On the basis of our previous work, young HBe-seroconverters generally experience fluctuations in ALT levels and early spontaneous HBe-SC at a peak age of 13–25 years,^6^ and delayed HBe-SC may occur after 26 years of age. Therefore, patients in each group were further subdivided into 3 age subgroups: 1–12 years, 13–25 years, and ≥26 years. Among the identified bacteria, only *R gnavus* and *A muciniphila* levels were affected by age. A high abundance of *R gnavus* was found primarily in children aged 1–12 years (Figure 1*C*), who are often in the IT state during this life period. In general, IT patients had a high titer of hepatitis B e antigen (HBeAg) with normal ALT levels, and an acute flare-up of ALT (>40 U/L) occurred as patients progressed toward IA status (Figure 1*C,* Table 1). In response to IA transition, the richness of *R gnavus* was diminished, and the constitution of the gut flora shifted to a predominance of *A muciniphila* in the 1–12- and 13–25-year-old age groups (Figure 1*C*). In this study, there were no patients with spontaneous HBe-SC in the 1–12-year-old age group (Figure 1*C*). Patients who experienced early spontaneous HBe-SC at 13–25 years showed rapid growth of *A muciniphila* during ALT flares, and the level of *A muciniphila* decreased after HBe-SC occurred (Figure 1*C*). In contrast, in patients who had delayed spontaneous HBe-SC at ≥26 years of age, the acquisition of a high frequency of *A muciniphila* did not occur at the IA stage but was postponed to the time when spontaneous HBe-SC was achieved (Figure 1*C*).

**Table 1:** Characteristics of Study Patients

	IT	IA
Case number	40	30
Male: female	14:26	13:17
Age (y)	1.2–58.1	1.7–45.6
Follow-up (y)	3.5	3.5
ALT (U/L), median (range)	21 (9–39)	61 (43–348)
HBeAg (IU/mL), median (range)	1522.66 (1124.773–1867.032)	1270.647 (5.419–1841.735)

Among these gut microbiota, *R gnavus* was unique in that it was the only bacterium that negatively associated with age, and the younger the individuals were, the more *R gnavus* inhabitation they exhibited (R = –0.2465, *P* < .03, Figure 1*D*). High *R gnavus* abundance in children (1–12 years old) was positively correlated with a longer IT duration (R = 0.5123, *P* < .02), whereas the abundance of *A muciniphila* was inversely associated with a shorter IT phase (R = –0.4264, *P* < .05, Figure 1*D*). Among the IT, IA, and spontaneous HBe-SC groups, a high fecal count of *A muciniphila* was further linked to elevated serum levels of ALT (R = 0.3389, *P* < .002) and decreased serum titers of HBeAg (R = –2445, *P* < .03, Figure 1*D*). In addition, increasing *A muciniphila* in IA patients was positively correlated with early HBe-SC (R = 0.5280, *P* < .003, Figure 1*D*), suggesting that *A muciniphila* may induce ALT flares to achieve IA transition and HBeAg loss to prompt HBe-SC. In contrast, *P copri*, *F umbilicate,* and *D formicigenerans* showed no correlation with clinical features (Figure 1*D*). Not only were the effects of *R gnavus* and *A muciniphila* evident in HBV infection (Figure 1*D*), but also their fecal levels in all CHB cases exhibited the opposite trends (Spearman rank correlation R = –0.4009, *P* < .0006, Figure 1*E*), perhaps attributed to their antagonistic roles in CHB progression.

### R gnavus and A muciniphila Impact the Course of HBV Infection

**Figure 2:**
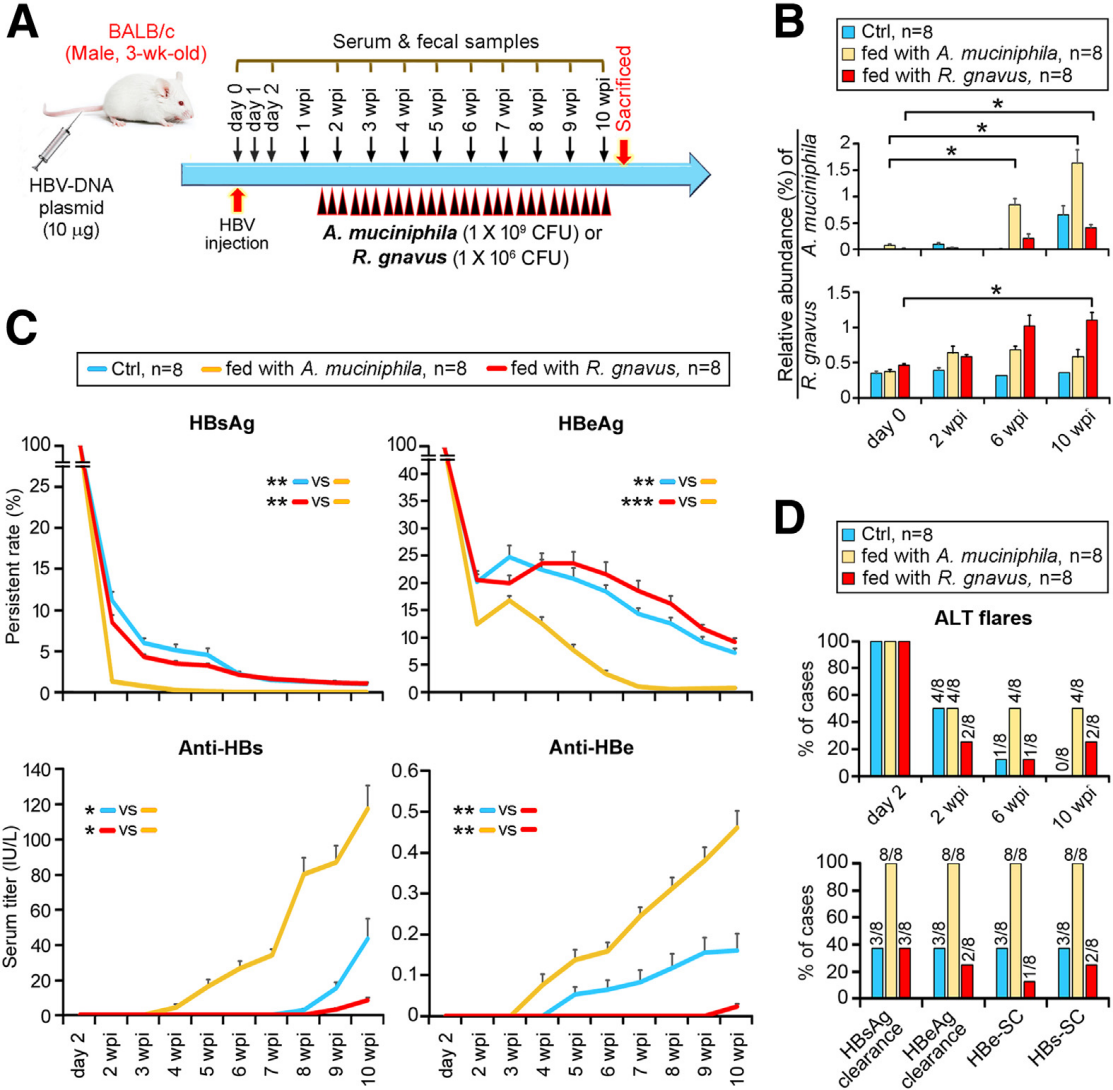
***A muciniphila* and *R gnavus* oppositely influence progression of HBV infection in BALB/c HBV-HDI mouse model.** (*A*) Schematic time schedule of the experiment. HBV-DNA1.2 plasmid was injected into the tail veins of mice. *A muciniphila* and *R gnavus* were anaerobically grown in modified GAM broth. These bacteria were intragastrically gavaged to mice 3 times/wk signified by *black arrowheads*, in which each of them represents 1 time of gavaging feed. Serum and fecal samples were collected at times indicated. Fresh fecal samples of day 0 were picked before HBV injection. Ctrl mice were gavaged with modified GAM broth only. (*B*) Fecal counts of *A muciniphila* and *R gnavus* (mean percentage of relative abundance ± SEM) were assessed by 16S-rRNA NGS. (*C*) Serial serum titers of HBsAg, HBeAg, anti-HBs, and anti-HBe antibodies (mean percentage of persistent rate ± SEM) were examined. (*D*) Histogram of percent of cases with ALT flares (≥50 U/L, *upper*), clearance of HBsAg and HBeAg, HBe-SC and HBs-SC at 10 wpi (*lower*). Student *t* test was applied to identify statistical significance. ∗*P* < .05, ∗∗*P* < .005, ∗∗∗*P* < .0005.

An HBV-HDI mouse model was established in this study to investigate the direct impact of *R gnavus* and *A muciniphila* on HBV infection and to provide a more comprehensive and dynamic representation of host-microbiota-virus interactions. BALB/c mice were adopted because of their propensity to sustain HBV infection.^5^ *A muciniphila* (ATCC BAA-835) or *R gnavus* (ATCC 29149) was intragastrically gavaged to these mice (male, 3 weeks old) after pAAV/HBV1.2 injection according to the schedule shown in Figure 2*A*. Active colonization of these bacteria was monitored by NGS profiling of fecal materials (Figure 2*B*). *A muciniphila*-fed mice had markedly reduced serum HBsAg/HBeAg and early production of antibodies to HBsAg (anti-HBs) and HBeAg (anti-HBe) compared with those of the control (Ctrl) mice (Figure 2*C*). In contrast, *R gnavus* mice exhibited a significantly retarded clearance rate of HBsAg/HBeAg and the production of anti-HBs/HBe (Figure 2*C*). Mice fed *A muciniphila* had a higher rate of ALT flares after 6 weeks post injection (wpi), followed by increased clearance of HBsAg and HBeAg up to 100% and complete achievement of HBe-SC and HBsAg seroconversion (HBs-SC) at 10 wpi, whereas *R gnavus* supplementation demonstrated the reverse impact (Figure 2*D*).

**Figure 3:**
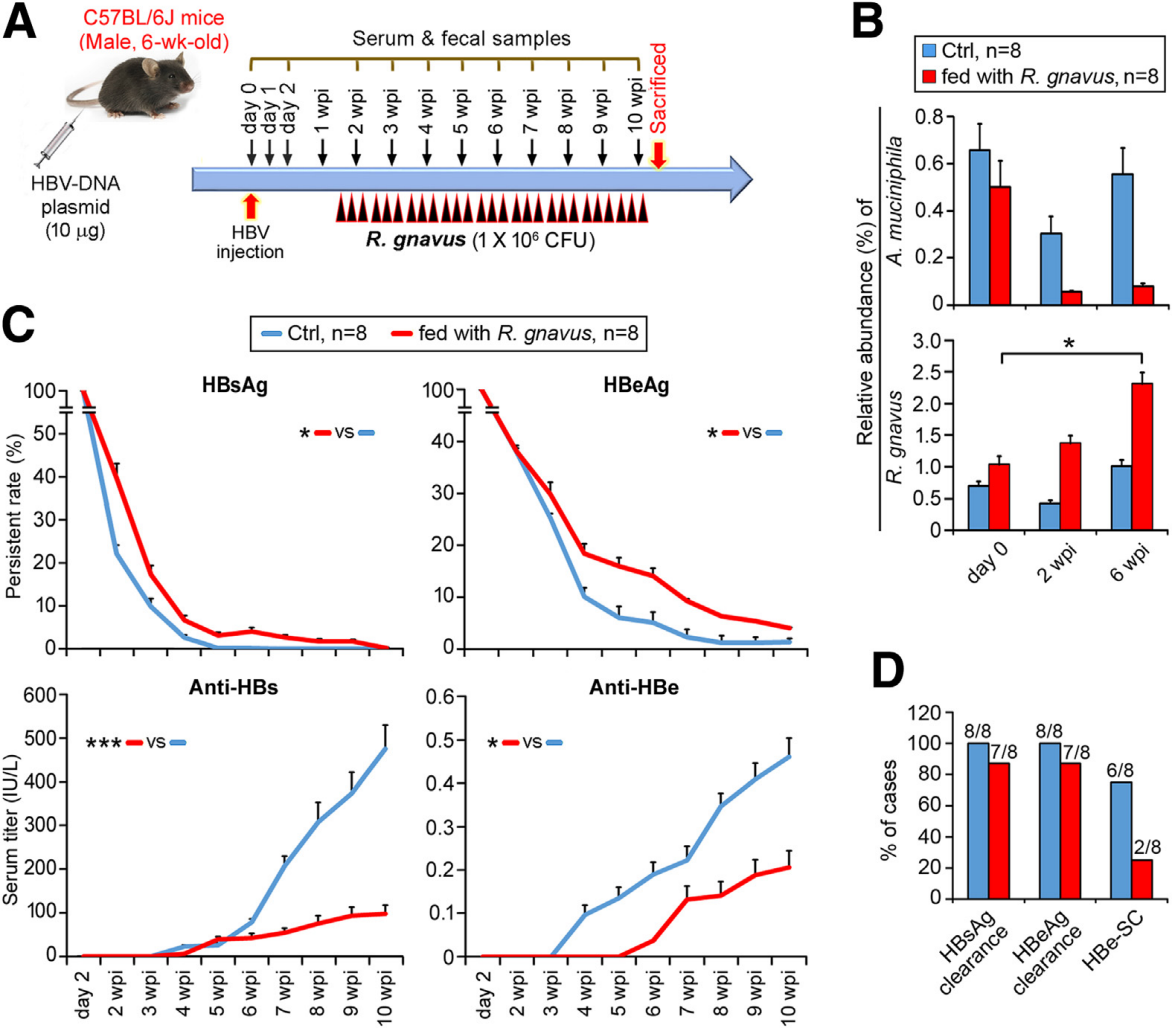
***R gnavus* promotes HBV persistence in C57BL/6J mice that predisposed to HBV infection.** (*A*) Schematic time schedule for generation of HBV-HDI mouse model. Mice (n = 8/group) were tail-vein injected with plasmid encoding HBV-DNA1.2. Live *R gnavus* was intragastrically gavaged to mice 3 times/wk as *arrowheads* indicate. Ctrl mice were gavaged with broth. Serum and fecal samples were collected at days 2 and 2–10 wpi, and fecal samples of day 0 were picked before HBV injection. (*B*) Fecal counts of *A muciniphila* and *R gnavus* were assessed by 16S rRNA NGS and reported as percentages of relative abundance ± SEM. (*C*) Serum levels of HBsAg, HBeAg, anti-HBs, and anti-HBe were examined at indicated time points and plotted as mean percentages of persistent rate ± SEM. Difference between groups was estimated with Student *t* test. ∗*P* < .05, ∗∗∗*P* < .0005. (*D*) Histogram of percentage of cases achieving clearance of HBsAg and HBeAg, as well as HBe-SC at 10 wpi. *Different color codes* symbolize groups of mice as described in *B*.

**Figure 4:**
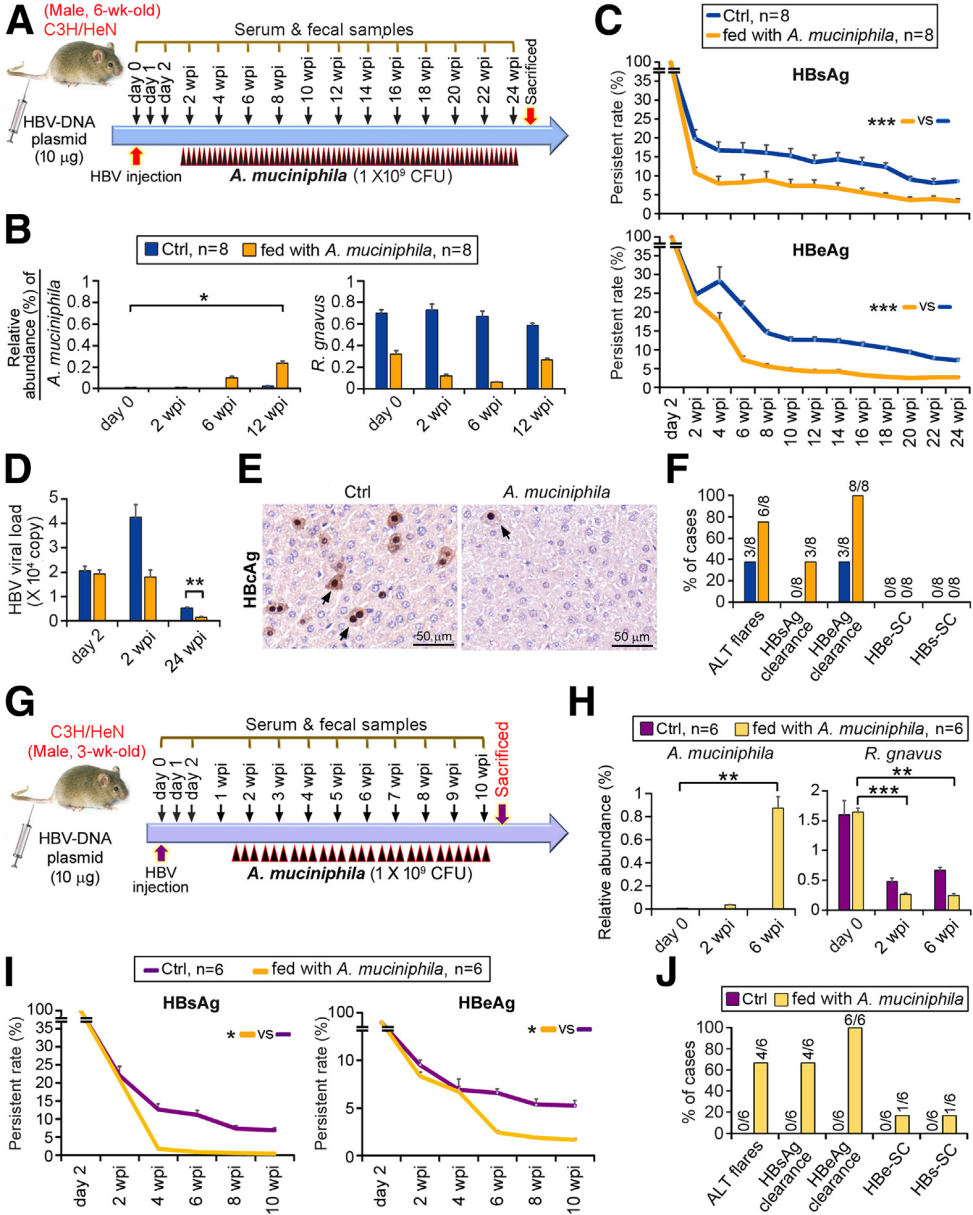
***A muciniphila* but not *R gnavus* changes the tendency of HBV chronicity toward rapid clearance.** (*A*) Schematic time schedule of establishment of C3H/HeN HBV-HDI mouse model (6-wk-old, n = 8/group). HBV-carried mice were developed by hydrodynamic injection of HBV-DNA1.2 plasmids into tail veins. Live *A muciniphila* was intragastrically gavaged to mice 3 times/wk during 1–24 wpi. *Black arrowhead* denotes each gavage time point. Ctrl mice were gavaged with broth. Serum and fecal samples were serially collected at times indicated. Fecal samples of day 0 were picked before HBV injection. (*B*) 16S rRNA NGS profiled and quantified fecal counts of *A muciniphila* and *R gnavus* (mean percentages of relative abundance ± SEM). (*C*) Serum levels of HBsAg and HBeAg (mean percentages of persistent rate ± SEM) were consecutively monitored. (*D*) Serum HBV viral load (mean copy number ± SEM) was measured by quantitative PCR. *Color codes* symbolize mice groups described in *B*. (*E*) Hepatic expression of HBcAg (*arrows*) was detected by immunohistochemistry. (*F*) Percentage of cases with ALT flares (detected at 6 wpi) and HBsAg/HBeAg clearance and HBe-SC/HBs-SC (detected at 24 wpi) was calculated. *Color codes* represent different mice groups as described in *B*. (*G*) Schematic time schedule of experiment established in young C3H/HeN HBV-HDI mouse model (3-wk-old, n = 6/group). Experiment protocol was scheduled as described above with only a slight modification wherein the duration was shortened to 10 wpi. (*H*) Fecal counts of *A muciniphila* and *R gnavus* were measured by 16S rRNA NGS and reported as percentages of relative abundance ± SEM. (*I*) Serum levels of HBsAg and HBeAg were examined at indicated time points, compared by Student *t* test, and plotted as mean percentages of persistent rate ± SEM. (*J*) Histogram of percentage of cases succeeded in clearance of HBsAg and HBeAg, as well as HBe-SC and HBs-SC, at 10 wpi. Data were evaluated by Student *t* test. ∗*P* < .05, ∗∗*P* < .005, ∗∗∗*P* < .0005.

To derive more evidence supporting the roles of *R gnavus* and *A muciniphila*, different mouse strains, such as C57BL/6J and C3H/HeN (Figures 3*A* and 4*A*), were tested. The former and the latter are representative of strains with fast and slow clearance of HBeAg, respectively.^5^ Before HBV infection (day 0), C57BL/6J mice were more prone to accommodate a higher rate of *A muciniphila* colonization than BALB/c and C3H/HeN mice (Figures 2*B,* 3*B,* and 4*B*), explaining the natural tendency of C57BL/6J mice to eradicate HBV infection. Administration of *R gnavus* to C57BL/6J mice led to delayed clearance of HBsAg/HBeAg, impeding anti-HBs/anti-HBe production and HBe-SC (Figure 3*C* and *D*). For C3H/HeN HBV-HDI mice, *A muciniphila* gavage was extended to 24 wpi to counteract the difficult eradication of HBeAg, and the active colonization of *A muciniphila* was monitored in comparison with the level of endogenous *R gnavus* (Figure 4*A* and *B*). Mice fed *A muciniphila* were more susceptible to eliminating HBV infection as reflected by the reduced serum levels of HBsAg/HBeAg/viral load and the expression of hepatic HBcAg (Figure 4*C–E*). In addition, *A muciniphila* mice showed high rate of ALT flares and achieved 100% and 42.9% of HBeAg and HBsAg clearance, respectively, at 24 wpi (Figure 4*F*). Considering that the maturation of the gut microbiota constitution is age dependent, we compared these 6-week-old C3H/HeN HBV-HDI mice with 3-week-old mice (Figure 4*G*). Both age groups showed negligible level of *A muciniphila* before HBV-HDI; moreover, 3-week-old mice had approximately 2-fold higher fecal counts of *R gnavus* (≈1.5%) than those of 6-week-old mice (≈ 0.7%, Figure 4*B* and *H*), resembling the high colonization of *R gnavus* in young CHB patients aged 1–12 years (Figure 1*B*). However, the level of *R gnavus* in 3-week-old mice was evidently reduced by *A muciniphila* feeding, and the clearance rates of HBsAg and HBeAg were therefore more rapid than those of 6-week-old mice (Figure 4*H*–*J*). This result may be due largely to the gut microenvironment of young 3-week-old C3H/HeN HBV-HDI mice effectively supporting the colonization of *A muciniphila*.

**Figure 5:**
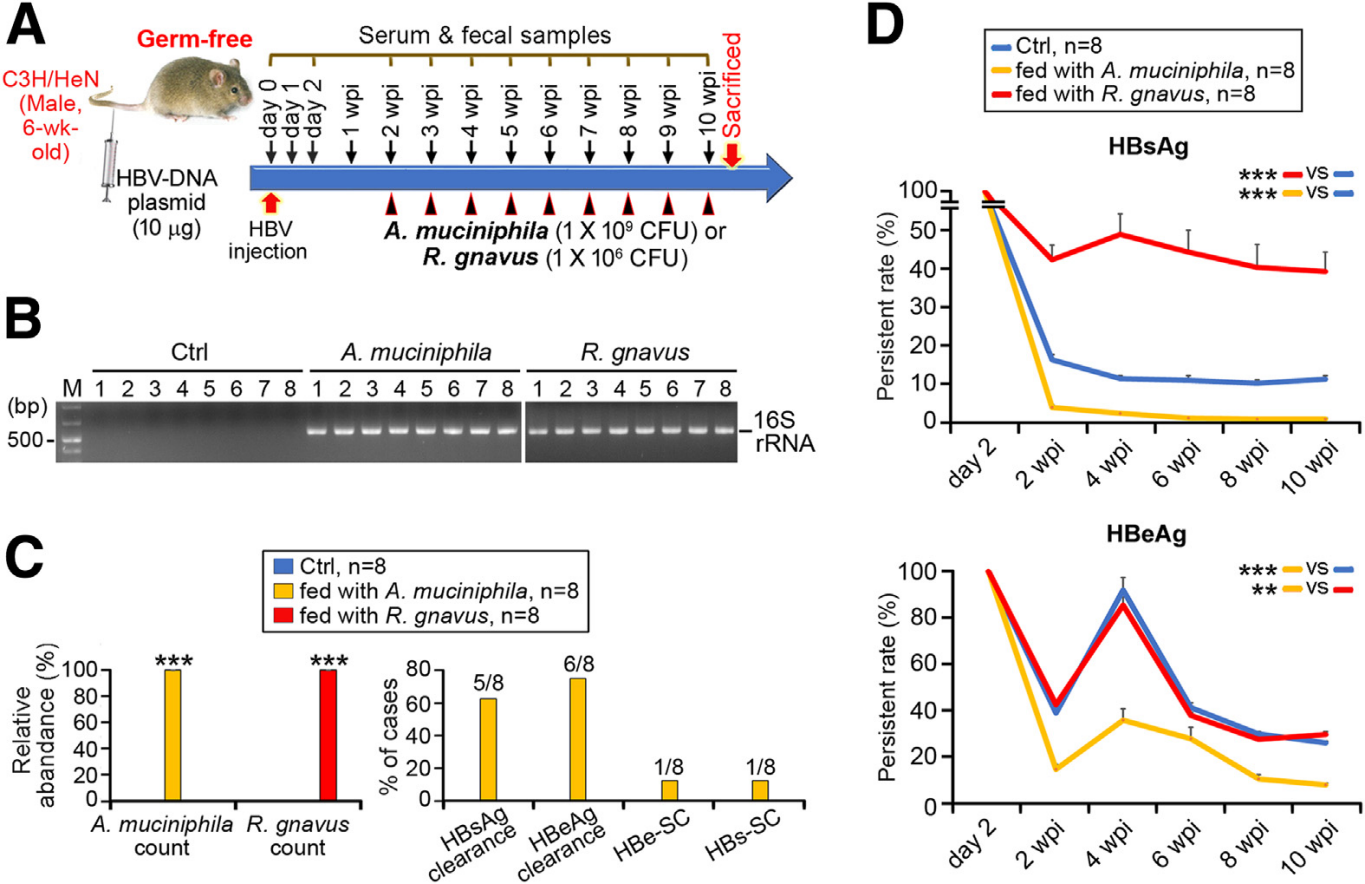
**HBV clearance, induced by *A muciniphila* but inhibited by *R gnavus,* in germ-free mice.** (*A*) Schematic time schedule for establishment of germ-free C3H/HeN HBV-HDI mice model. Each *black arrowhead* represents 1 time point of *A muciniphila/R gnavus* administration, and frequency of gavaging was 1 time/wk. (*B*) PCR targeting V3 and V4 16S rRNA hypervariable regions was conducted on stool samples harvested at 10 wpi. (*C*) 16S rRNA NGS quantified the fecal counts of *A muciniphila* and *R gnavus* (mean percentages of relative abundance ± SEM). Student *t* test was used to test for differences between Ctrl and *A muciniphila/R gnavus*-treated mice (∗∗∗*P* < .0005, *left*). Percentage of cases achieving clearance of HBsAg and HBeAg, HBe-SC and HBs-SC was assessed (*right*). (*D*) Serum HBsAg and HBeAg (mean percentages of persistent rate) of germ-free C3H/HeN HBV-HDI mice were time-serially measured. Data were evaluated by Student *t* test. ∗*P* < .05, ∗∗*P* < .005, ∗∗∗*P* < .0005.

To consolidate the roles of *R gnavus* and *A muciniphila* in HBV infection, germ-free C3H/HeN mice were fed with these bacteria post-HBV-HDI (Figure 5*A*). 16S rRNA-specific polymerase chain reaction (PCR) and NGS both confirmed the successful monocolonization of *R gnavus* and *A muciniphila* in these germ-free mice (Figure 5*B* and *C*, left). *A muciniphila* germ-free mice showed quick removal of serum HBsAg/HBeAg, whereas these antigens were still highly expressed in the sera of *R gnavus*-fed germ-free mice (Figure 5*D*). Also, *A muciniphila*-fed germ-free mice tended to elicit HBsAg/HBeAg clearance at 10 wpi, which was faster than that in normal C3H/HeN mice (24 wpi, Figures 4*F* and 5*C*, right). This verified that *A muciniphila* alone could eliminate HBV infection without any assistance from other commensal bacteria.

### Progression of CHB Is Linked to Changes in Cholesterol Metabolism

**Figure 6:**
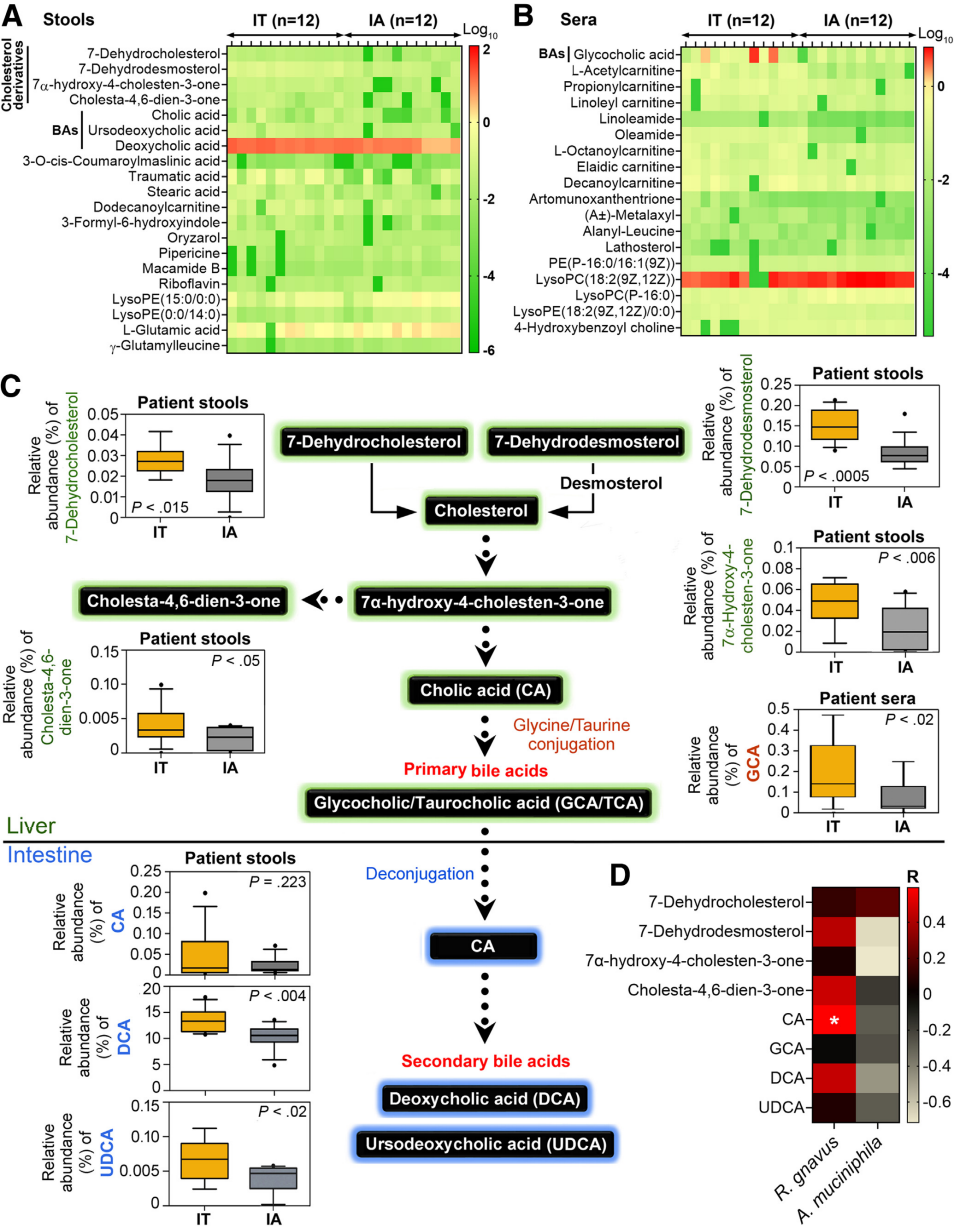
**Metabolomic changes during disease progression of CHB.** (*A* and *B*) Stools (*A*) and sera (*B*) samples collected from IT and IA patients were subjected to UHPLC-Orbitrap-MS–based untargeted metabolite analysis. Weighted heat map images were constructed according to the log_10_-transformed abundance of metabolites. The top 20 metabolites identified from stool samples (*A*) and top 18 from sera (*B*) are shown. (*C*) Cholesterol biosynthesis and metabolism pathway are schematically shown. Results of UHPLC-Orbitrap-MS–quantified metabolites are depicted as *box plots* and analyzed by Student *t* test. (*D*) Spearman correlation coefficients assessing relationships between relative amounts of gut microbiota and the metabolites identified. All the indicated metabolites were isolated from fecal samples of IT and IA patients except GCA, which was extracted from their serum samples.

To unravel the mechanism of how *R gnavus* and *A muciniphila* are involved in HBV infection, CHB patients’ fecal and serum samples were analyzed using ultra-high-performance liquid chromatography coupled with Orbitrap mass spectrometry (UHPLC-Orbitrap-MS) to obtain an untargeted metabolite profile. The top 20 fecal and top 18 serum variables with statistical significance were categorized in hierarchical clustering heat maps (Figure 6*A* and *B*). Cholesterol derivatives and bile acids (BAs) were the most apparent metabolites that distinguished IT patients from IA patients, with higher amounts in the stool and sera of IT patients (Figure 6*A* and *B*). KEGG annotation revealed that these relevant metabolites were the biosynthetic precursors (7-dehydrocholesterol and 7-dehydrodesmosterol) and intermediates (cholesta-4,6-dien-3-one and 7α-hydroxy-4-cholesten-3-one) of cholesterol metabolism (Figure 6*C*). 7α-Hydroxy-4-cholesten-3-one is then bioconverted to cholic acid (CA), which can enter the blood circulation or be excreted into the intestine after conjugation with glycine and taurine to form primary BAs (glycocholic acid and taurocholic acid).^7^^,^^8^ Intestinal microbes deconjugate the primary BAs, liberating CA from the conjugated BAs and transforming them into secondary BAs (deoxycholic acid [DCA] and ursodeoxycholic acid [UDCA]).^7^ The levels of all these metabolites except CA were significantly decreased in the sera and stool samples of IA patients compared with those of the IT group (Figure 6*C*). Instead, CA was markedly correlated with the level of fecal *R gnavus* in the IT and IA groups (Spearman correlation R = 0.4923, *P* < .04, Figure 6*D*), hinting at an enrichment of CA by *R gnavus*.

### R gnavus and A muciniphila Intervene in CA Biosynthesis to Control HBV Infection

**Figure 7:**
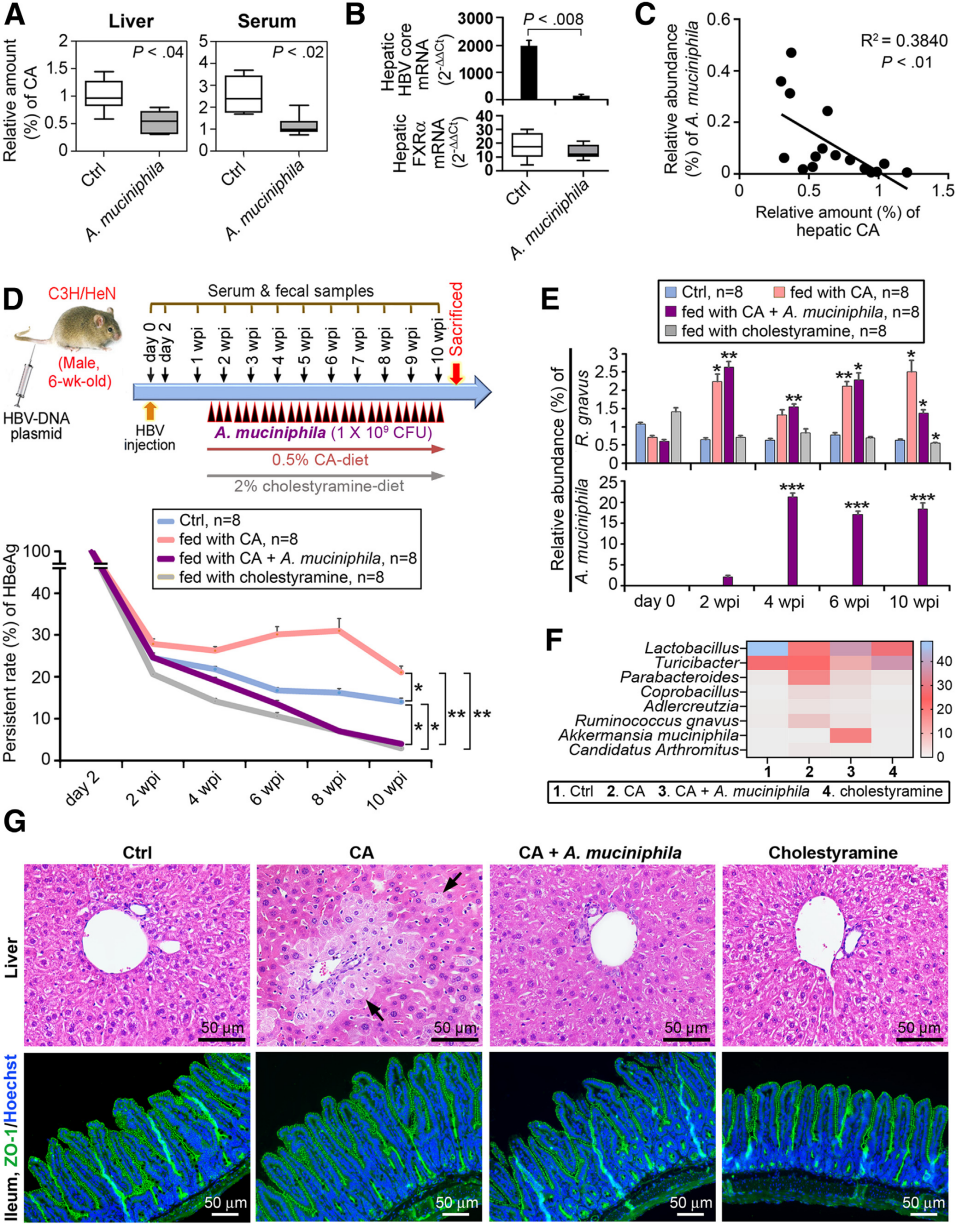
**Reverse regulation between *A muciniphila* and *R gnavus* in CA biosynthesis.** (*A*) UHPLC-Orbitrap-MS–based untargeted metabolite analysis was achieved on liver tissues and sera samples of C3H/HeN HBV-HDI mice fed with modified GAM broth (Ctrl) or *A muciniphila* (n = 8/group). *Box plots* showed the differences of CA scores. (*B*) Quantitative PCR analysis of mRNA levels of HBV core (*upper*) and FXRα (*lower*) in liver tissues. Changes in expression levels were determined by 2^−ΔΔCt^. (*C*) Linear regression analysis revealed the correlation between fecal *A muciniphila* and hepatic CA detected at 24 wpi. (*D*) Schematic time schedule of the study protocol. Mice were fed daily with either 0.5% CA or 2% cholestyramine diets at the same administration time as indicated. *A muciniphila* was intragastrically gavaged to CA-feeding mice 3 times/wk as specified by *black arrowheads*. Serum HBeAg was plotted as mean percentage of persistent rate ± SEM. (*E*) Fecal counts of *R gnavus* and *A muciniphila* were determined by 16S rRNA NGS. Results of 2–10 wpi were compared with the respective values of 0 wpi. Significant differences between groups depicted in *D* and *E* were estimated by Student *t* test. ∗*P* < .05, ∗∗*P* < .005, ∗∗∗*P* < .0005. (*F*) Heat map depicting the top 8 bacteria identified by NGS analysis using fecal samples of indicated mice collected after sacrifice. *Color code scale* shows relative abundance (%) of bacteria indicated. (*G*) Histologic features detected by H&E staining (*upper*) on liver tissues of mice fed with CA, CA+*A muciniphila,* and cholestyramine. *Black arrows* denote the necrotic hepatocytes surrounding the portal area. ZO-1 (*green*)-specific immunofluorescence assay (*lower*) was performed on ileum tissue sections of mice indicated. Hoechst fluorescence (*blue*) displays distribution of nuclei.

The CA level was relatively low in the livers and sera of *A muciniphila*-fed C3H/HeN HBV-HDI mice compared with Ctrl mice (Figure 7*A*), recapitulating the reduction of CA in IA patients whose *A muciniphila* content was augmented (Figure 6*C*). CA is one of the ligands of farnesoid X receptor alpha, a liver-enriched nuclear receptor, which is loaded onto the core promoter of HBV to drive core (HBcAg) transcription.^9^ After this, we observed a concurrent down-regulation of CA and farnesoid X receptor alpha in the livers of *A muciniphila* mice, thus decreasing the hepatic expression of HBcAg mRNA (Figure 7*A* and *B*). In addition, a negative correlation between hepatic CA and fecal *A muciniphila* levels was documented for all these mice at 24 wpi (Figure 7*C*), indicating *A muciniphila* blocked the transcription of HBcAg by suppressing CA levels.

To elaborate the role of CA in HBV persistence, we fed the C3H/HeN HBV-HDI mice a 0.5% CA diet (Figure 7*D*). As a result, a high persistence rate of HBeAg was demonstrated upon CA supplementation and gave rise to the growth of *R gnavus* (Figure 7*D*–*F*). However, mice gavaged with *A muciniphila* eliminated HBeAg from the sera and exhibited a diminished richness of *R gnavus* regardless of CA consumption (Figure 7*D*–*F*), suggesting that *A muciniphila* prevented CA-driven HBV persistence via inhibition of *R gnavus*. More notably, mice nourished with a diet containing cholestyramine, which is a BA sequestrant,^10^ exhibited expedited HBeAg clearance and inhibited growth of *R gnavus* (Figure 7*D*–*F*), indicating that *R gnavus*–associated HBV persistence could be alleviated by BAs scavenging or *A muciniphila* intake. CA administration not only altered the commensal homeostasis of the gut microbiota by switching the microbial community toward pathogenic bacteria, such as increasing the *Parabacteroides* (BSL2 bacteria) abundance and decreasing the richness of probiotic *Lactobacillus*, but also caused hepatic necrosis (Figure 7*F* and *G*, upper). Yet, CA intake did not disrupt the intact intestinal epithelial barrier, as evidenced by Zonula Occludens-1 (ZO-1) staining (Figure 7*G*, lower). A noteworthy feature was that the CA-induced bacterial imbalance and liver injury could be ameliorated by treatment with *A muciniphila* (Figure 7*F* and *G*).

**Figure 8:**
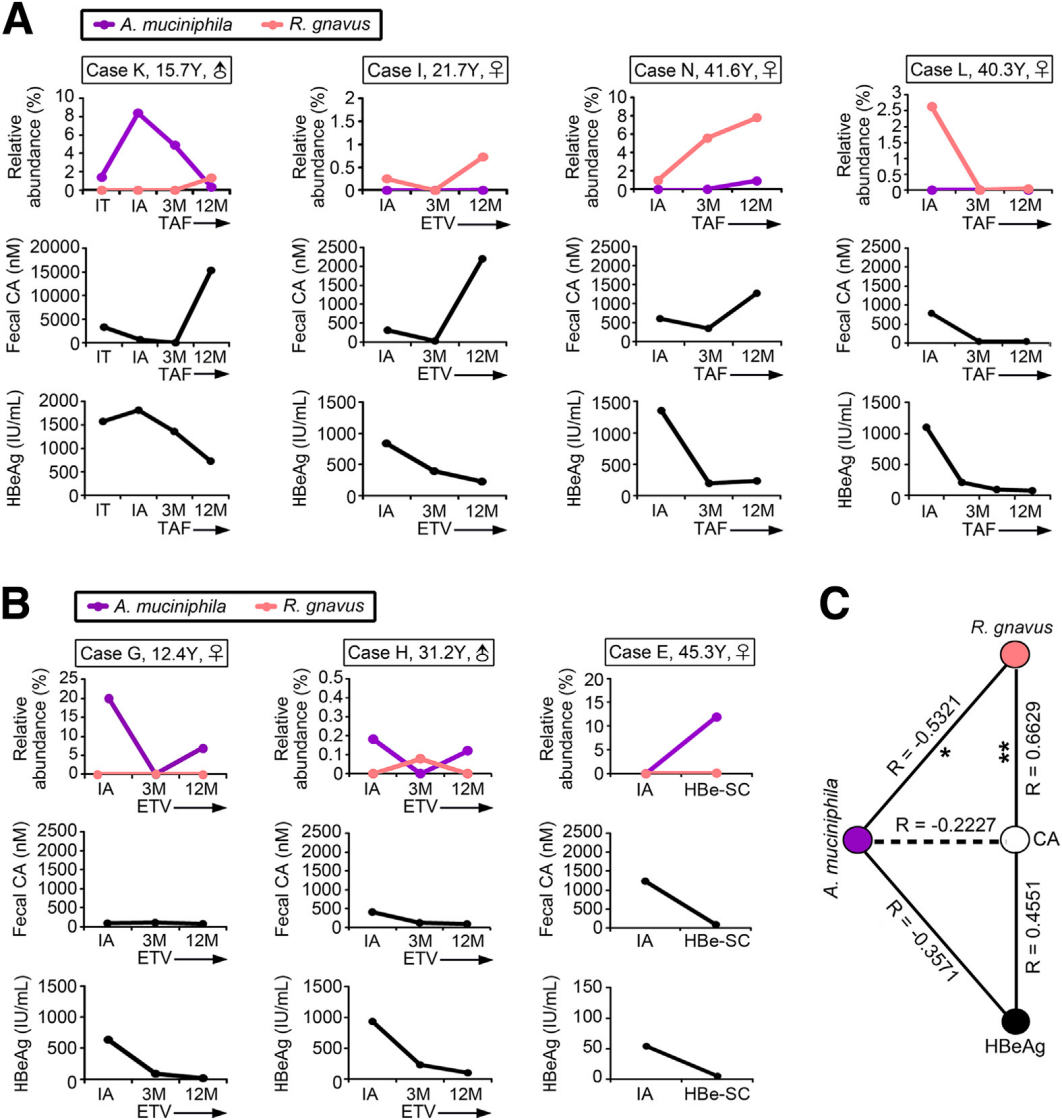
**Cross-correlation time-series analysis of fecal CA and serum HBeAg in accordance with abundance of *R gnavus* and *A muciniphila*.** (*A* and *B*) Serial sampling was conducted to obtain stool and serum samples from CHB patients who received ETV or TAF therapy, as well as patients who experienced HBe-SC. M, months. Fecal counts of bacteria were analyzed by 16S rRNA NGS, and fecal CA level was measured by targeted BAs liquid chromatography coupled to tandem mass spectrometry and serum titer of HBeAg by immunoassay. Patients who showed positive correlation between *R gnavus* and fecal CA are presented in *A*, and those who demonstrated negative correlation between *A muciniphila* and fecal CA are displayed in *B*. (*C*) Spearman correlation was estimated using serial sampling data of *A* and *B* for revealing relationships among *A muciniphila*, *R gnavus*, fecal CA, and serum HBeAg. ∗*P* < .03, ∗∗*P* < .002.

Further evidence from a human study revealed a positive correlation between the fecal levels of *R gnavus* and CA in CHB patients after tenofovir alafenamide fumarate (TAF) or entecavir (ETV) treatment (Figure 8*A*). As seen in cases K, I, and N, the growth of *R gnavus* was associated with an increase in fecal CA that perturbed HBeAg clearance, and the reduction in *R gnavus* in case L diminished the levels of CA and HBeAg (Figure 8*A*). In contrast, increasing the *A muciniphila* abundance in cases G and H after ETV management, as well as case E who experienced spontaneous HBe-SC, not only constrained the growth of *R gnavus* but also limited the yield of CA, resulting in the fast eradication of HBeAg (Figure 8*B*). These data implied that the down-regulation of CA was dependent on the blooming of *A muciniphila* but not on anti-HBV drugs. It appeared that a high abundance of *R gnavus* tended to boost CA accumulation (Spearman correlation R = 0.6629, *P* < .002, Figure 8*C*). The weak negative relationships between *A muciniphila* and CA (Spearman correlation R = –0.2227, *P* = .3594) and *A muciniphila* and HBeAg (Spearman correlation R = –0.3571, *P* = .4444) might be an indirect effect, perhaps resulting from the antagonism between *A muciniphila* and *R gnavus* (Spearman correlation R = –0.5321, *P* < .03, Figure 8*C*).

### Antagonistic Effect Between R gnavus and A muciniphila Reprograms Cholesterol Metabolism

**Figure 9:**
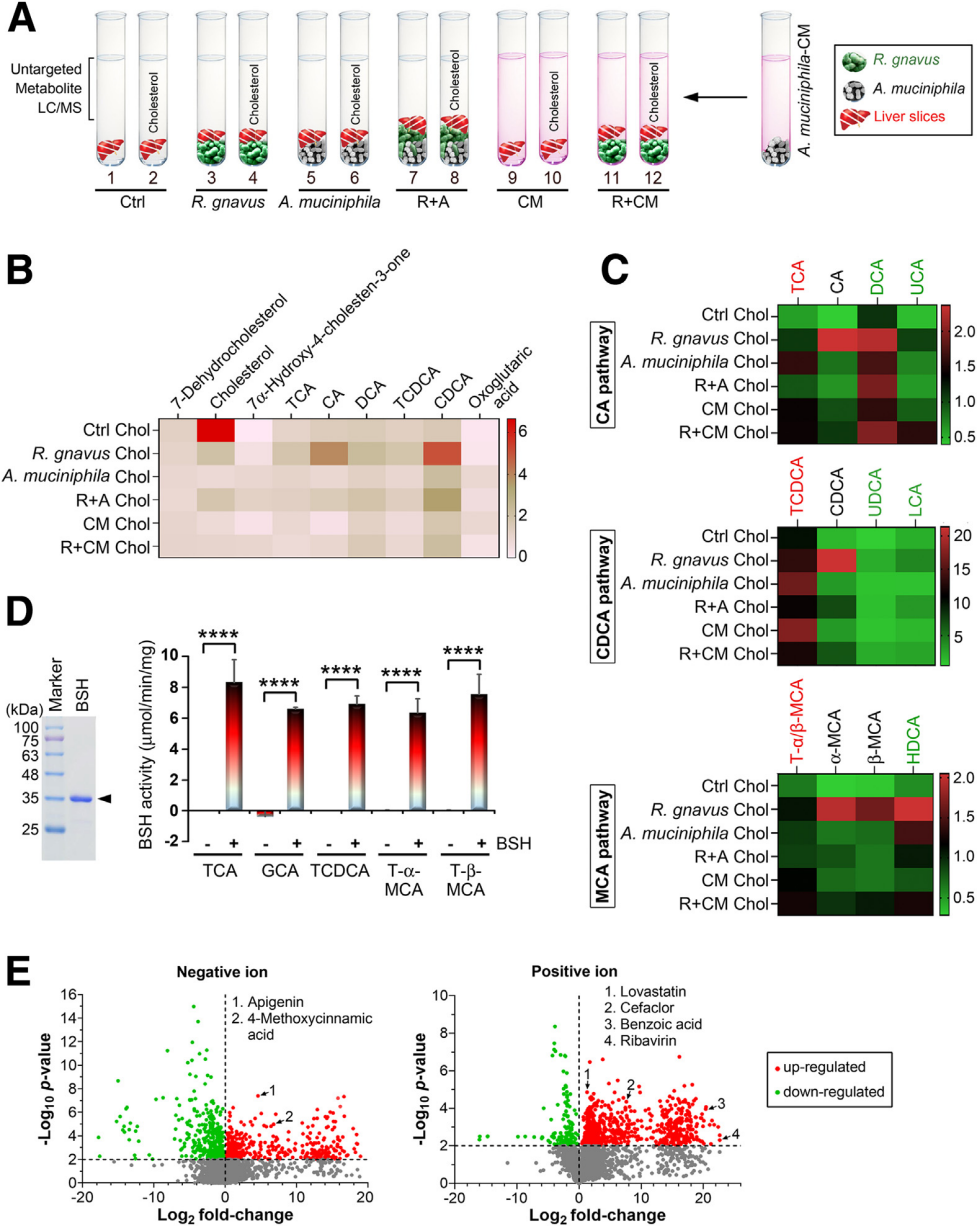
***A muciniphila* obstructs the BAs bioconversion activity of *R gnavus*.** (*A*) Schematic diagram representing the in vitro cholesterol metabolism assay. Each reaction contained 65 mg liver slices in 3 mL modified GAM medium with or without *R gnavus* (2 × 10^7^), *A muciniphila* (2 × 10^10^), and 1 mmol/L cholesterol. Reactions (No. 9–12) contained *A muciniphila*-CM, which was prepared by centrifuging *A muciniphila* culture at 15,000*g* for 20 minutes and passed through a 0.22-μm pore size filter to fully remove bacterial cells. (*B*) UHPLC-Orbitrap-MS–based untargeted metabolite analysis was performed on supernatant obtained from *A*. Result was shown as fold relative to respective reactions without cholesterol supplement. Only a subset of metabolites that have significant fold changes are selected for visualization in a heat map. A, *A muciniphila*; Chol, cholesterol; CM, *A muciniphila*-CM; R, *R gnavus*. (*C*) Heat map clustering analysis constructed on basis of fold change in targeted BAs liquid chromatography coupled to tandem mass spectrometry profiling. Culture supernatants depicted in *A* were subjected to this assay. Primary and secondary BAs are highlighted in *red* and *green fonts*, respectively. (*D*) In vitro BSH enzymatic activity was measured using TCA, GCA, TCDCA, T-α-MCA, and T-β-MCA as substrates in the presence or absence of BSH of *R gnavus*. Protein expression of BSH was confirmed by Coomassie blue-stained sodium dodecyl sulfate–polyacrylamide gel electrophoresis analysis. (*E*) UHPLC-Orbitrap-MS characterization of *A muciniphila*-CM. *Volcano diagrams* displayed up-regulated (*red*) and down-regulated (*green*) metabolites that identified by negative and positive ion modes after normalized to the modified GAM control broth. Metabolites showed greatest value on the x-axis, which were found only in the *A muciniphila-*CM but completely absent from the GAM broth.

An in vitro cholesterol metabolism assay was established to prove that *R gnavus* and *A muciniphila* regulate HBV infection by directly manipulating CA bioconversion without other microbial involvement. These bacteria were cultured in modified GAM medium. Conditional medium (CM) of *A muciniphila* (*A muciniphila*-CM) containing bacterially derived metabolites was prepared. Cholesterol was used as a metabolism source. Liver slices obtained from 6-week-old C3H/HeN mice were incubated in all reactions to provide enzymes required for cholesterol metabolism (Figure 9*A*). After 24 hours of culture under 37°C anaerobic conditions, the supernatant was collected for UHPLC-Orbitrap-MS–based untargeted metabolite analysis, and the results were depicted in a heat map after standardization with the corresponding reactions without cholesterol. 7-Dehydrocholesterol, which existed in the liver slices, was equally abundant among all reactions (Figure 9*B*), indicating that each reaction has a nearly identical liver input. Upon incubation with *R gnavus* and/or *A muciniphila*, the cholesterol level was markedly reduced as compared with the Ctrl reaction without bacteria (Figure 9*B*). 7α-Hydroxy-4-cholesten-3-one was absent in the Ctrl reaction because cholesterol metabolism did not occur unless *R gnavus* and/or *A muciniphila* was administered (Figure 9*B*). In the reaction containing *R gnavus*, cholesterol was rapidly transformed into CA and chenodeoxycholic acid (CDCA, Figure 9*B*). Although *A muciniphila* alone did not influence the bioconversion of CA and had a limited effect on CDCA production, it suppressed the *R gnavus*–mediated biosynthesis of CA and CDCA during co-culture (Figure 9*B*), hinting at a direct cholesterol removal ability of *A muciniphila*. Notably, *A muciniphila*-CM treatment achieved an effect that was comparable with that of live *A muciniphila* (Figure 9*B*), highlighting that *A muciniphila* may degrade cholesterol via its secretory compounds. The level of oxoglutaric acid, one of the Krebs cycle intermediates for energy generation, was slightly increased in the presence of *A muciniphila* and *A muciniphila*-CM during cholesterol metabolism (Figure 9*B*).

To elucidate the BA species regulated by *R gnavus* and *A muciniphila*, culture supernatants of this in vitro cholesterol metabolism assay were further applied for targeted BAs liquid chromatography coupled to tandem mass spectrometry measurements. An enrichment of unconjugated BAs (CA, CDCA, and α/β-MCA) and certain secondary BAs (DCA and hyodeoxycholic acid [HDCA]) was found in the presence of *R gnavus* (Figure 9*C*). In line with the observation from untargeted metabolite analysis (Figure 9*B*), neither live *A muciniphila* cells nor *A muciniphila*-CM could transform cholesterol into primary and secondary BAs, but both obstructed this function of *R gnavus* during co-cultivation (Figure 9*C*).

All primary BAs are deconjugated by bile salt hydrolase (BSH) to release free glycine and taurine.^7^ We assumed that *R gnavus* may express BSH to enrich uncojugated BAs. A *BSH* gene (977 bp, GeneID 57432353) encoding 325 amino acids was identified from the genome of *R gnavus* ATCC 29149. This gene was cloned and expressed in an *Escherichia coli* expression system for enzymatic analysis. When incubated with primary conjugated BAs (TCA, GCA, TCDCA, T-α-MCA, and T-β-MCA), which were used as substrates, *R gnavus* BSH displayed efficient hydrolysis activity to remove taurine and glycine from these primary BAs, and the amounts of liberated taurine and glycine representing the functional activity of BSH could be detected by the ninhydrin test (Figure 9*D*).

*A muciniphila*-CM was subjected to untargeted metabolomics profiling of the excretory-secretory products of *A. muciniphila* using UHPLC-Orbitrap Elite/MS–based data acquisition. In comparison with the modified GAM control broth, 2139 up-regulated and 1757 down-regulated secretome metabolites of *A muciniphila*-CM were identified in negative ion mode (Figure 9*E*). In addition, 5045 up-regulated and 2276 down-regulated metabolites were revealed in positive ion mode (Figure 9*E*). Most of the metabolites were unidentified in the tandem mass spectrometry libraries. However, 6 metabolites that exhibited structural similarities to clinically used compounds/drugs (apigenin, 4-methoxycinnamic acid, lovastatin, cefaclor, benzoic acid, and ribavirin) seemed to be potentially important for *A muciniphila* to antagonize the function of *R gnavus* (Figure 9*E*). Apigenin and lovastatin can reduce total cholesterol in the serum.^11^^,^^12^ Lovastatin increases Krebs cycle flux by augmenting the concentration of Krebs cycle intermediates,^13^ explaining the capability of *A muciniphila*-CM to elevate the level of oxoglutaric acid (Figure 9*B*). In addition, apigenin, 4-methoxycinnamic acid, cefaclor, and benzoic acid possess antibacterial activities.^14^, ^15^, ^16^, ^17^ Furthermore, apigenin and ribavirin are potent antiviral agents with broad-spectrum activity against a wide variety of viruses including hepatitis C virus (HCV).^18^^,^^19^ All these identified metabolites indicated that *A muciniphila*-CM may have cholesterol-lowering, antibacterial, and antiviral properties.

### A muciniphila-CM Suppresses the Multiplication of R gnavus and HBV

**Figure 10:**
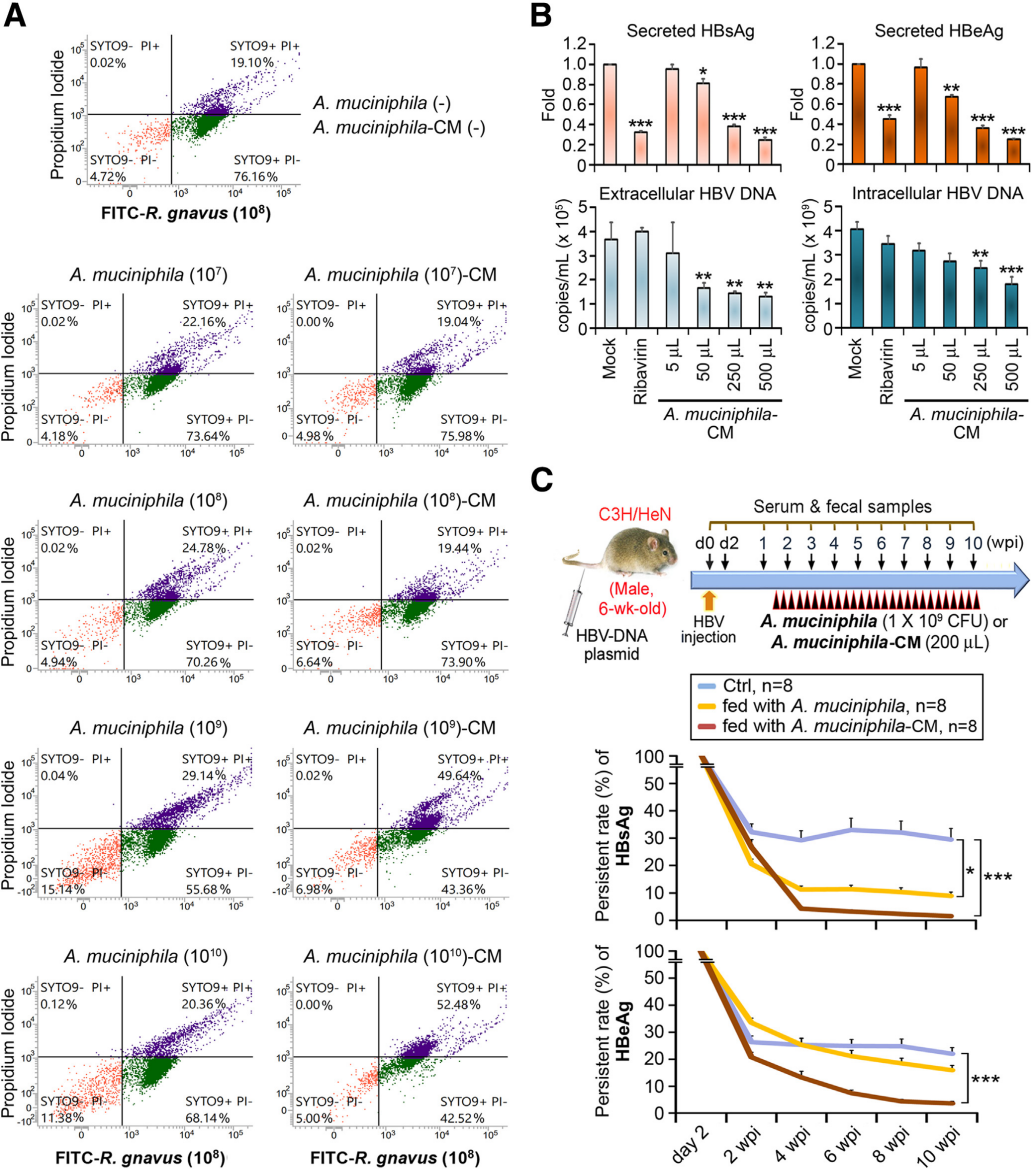
***A muciniphila*-CM eliminates HBV infection through its antibacterial and antiviral activities.** (*A*) Flow cytometry assessing survival of *R gnavus* on co-cultivation with *A muciniphila* live cells or *A muciniphila*-CM for 24 hours. After performing propidium iodide stain, live (*green*) and dead (*purple*) FITC-*R gnavus* were analyzed. (*B*) Anti-HBV activity of *A muciniphila*-CM was measured by co-culturing with HBV-transfected Huh-7 cells in comparison with ribavirin (800 μmol/L) treatment. Culture media were harvested for detecting the secreted HBsAg, HBeAg, and extracellular and intracellular HBV-DNA 24 hours after treatment. Culture media were collected for measurement of extracellular HBV-DNA. Cultured cells were lysed for assessing intracellular HBV-DNA. (*C*) Schematic time schedule for establishing HBV-HDI mice administered with *A muciniphila* and *A muciniphila*-CM. Gavaging was performed 3 times/week as specified by *black arrowheads with red rim*. Day 0 (time of HBV injection) and day 2 are denoted by d0 and d2, respectively. Serum levels of HBsAg and HBeAg of each group were measured (mean percentages of persistent rate ± SEM). Student *t* test. ∗*P* < .05, ∗∗∗*P* < .0005.

To test the antibacterial activity of *A muciniphila*-CM, *R gnavus* was subjected to green fluorescent SYTO 9 nucleic acid staining. Fluorescent *R gnavus* was co-cultured with increasing numbers of live *A muciniphila* or their respective CM for 24 hours and then subjected to propidium iodide staining for viability determination. Live *R gnavus* with intact cell membranes are represented in green, whereas dead *R gnavus* with damaged membranes and propidium iodide positivity are represented in purple. It was clear that increasing *A muciniphila* live cells did not obviously threaten the survival of *R gnavus*, yet *A muciniphila*-CM did (Figure 10*A*).

The antiviral activity of *A muciniphila*-CM was assessed by treating HBV-transfected Huh-7 cells, an HCC cell line, with increasing doses of *A muciniphila*-CM in comparison with ribavirin. After 24 hours of *A muciniphila*-CM incubation, the contents of HBsAg and HBeAg that released into the culture media were decreased in a dose-dependent manner and even more so than those with ribavirin administration (Figure 10*B*). Furthermore, *A muciniphila*-CM successfully reduced the extracellular and intracellular HBV DNA, but ribavirin failed to do so (Figure 10*B*). *A muciniphila*-CM exhibited antibacterial and antiviral activities against *R gnavus* and HBV; therefore, we compared its ability with that of live *A muciniphila* in combating HBV infection in an HBV-HDI mouse model. *A muciniphila*-CM was separated from its live cells by centrifugation and filtration. Then, live cells were washed and resuspended in fresh medium and gavaged into the mice. Mice nourished with *A muciniphila*-CM demonstrated an improved efficiency in HBsAg and HBeAg clearance that was superior to that in mice fed with live *A muciniphila* (Figure 10*C*), confirming the importance of secretory metabolites of *A muciniphila* in the treatment of CHB.

Collectively, this study demonstrated that 2 indigenous gut bacteria have opposite effects on the progression of CHB. *R gnavus* promotes HBV persistence, whereas *A muciniphila* eradicates HBV. *R gnavus* deconjugates primary BAs by its BSH product and governs BA metabolism to switch the host immune system toward prolonging HBV infection, yet *A muciniphila* counteracts this mechanism using its secretory metabolites.

## Discussion

The causes of undulant ALT flare-ups in the clinical course of CHB remain largely elusive. Factors that trigger ALT flares subsequent to HBe-SC are unidentified. We explored this puzzle with a brand new approach, that is, from a gut microbiota viewpoint. We demonstrated the antagonism between *A muciniphila* and *R gnavus* represents a double-edged sword in regulating HBV infection. A high abundance of *R gnavus* perturbed HBe-SC, whereas *A muciniphila* triggered HBe-SC. *R gnavus* mediated CA biosynthesis to promote HBV persistence through its encoded BSH, and *A muciniphila* counteracted this effect via its natural antibacterial and antiviral products to drive ALT flares and the loss of HBV antigens. These novel findings will certainly confer a groundbreaking impact on the future therapy of CHB because the microbial metabolites may have therapeutic benefits, and *R gnavus* and BAs may become new therapeutic targets for CHB patients.

According to American Association for the Study of Liver Diseases and Asian Pacific Association for the Study of the Liver guidelines, the diagnostic criteria for an IT phase include not only the positivity of HBeAg and normal ALT but also absence of liver injury or minimal steatosis/fibrosis for patients >35 years of age. Thus, all patients of ≥26-years age group received FibroScan assessment before categorizing as IT phase. For patients aged <35 years, the stage of fibrosis is not requisite for IT diagnosis. Thus, FibroScan test was not conducted in patients of 1–12-year and 13–25-year age groups. Using these diagnostic classifications, most young children who met the IT criteria acquired an abundance of *R gnavus* colonization, explaining why children with CHB are usually difficult to initiate the breakthrough of IT phase. As *A muciniphila* increased in abundance later in life, there was a chance for immune clearance to occur.

*R gnavus* is a Gram-positive intestinal symbiont associated with a series of inflammatory disorders such as inflammatory bowel disease^20^^,^^21^ and allergic diseases.^22^ Recent works have pointed to an important link between *R gnavus* and liver pathology, wherein an increase in the fecal count of *R gnavus* is predictive of the progression of nonalcoholic fatty liver disease to cirrhosis.^23^ In addition, it represents an important biomarker taxon in the tumor regions of HCC patients who had been infected by HBV and/or HCV.^24^^,^^25^ *A muciniphila*, a Gram-negative obligate anaerobe, has been proven to be a promising next generation probiotic, because it confers positive health benefits in ameliorating obesity,^26^ diabetes,^27^ hepatic steatosis,^28^ alcoholic liver disease,^29^ and liver injury.^30^ *A muciniphila* generally presents a better therapeutic effect because it protects the gut barrier, enhances mucus thickness, and reduces bacterial translocation, resulting in the reshaping to the gut microbiota constitution and immune responses toward good prognosis.^26^^,^^29^^,^^30^

*Akkermansia* is diminished upon establishment of HBV infection in C57BL/6J mice but returns to normal levels after ETV therapy; moreover, the richness of *Akkermansia* is negatively correlated with serum and hepatic HBV DNA load.^31^^,^^32^ Although the ebb and flow of *Akkermansia* had been noticed during HBV infection, the capture sequencing data of this bacterium are recognized only at the genus level during taxonomic classification. Therefore, studies have failed to accurately comprehend the exact species and the precise mechanism that correspond to the disease. Our work identified the key bacterial species, *A muciniphila* and *R gnavus*, in CHB pathogenesis.

*A muciniphila*-CM was a potent antagonist of *R gnavus* survival and activities including BA biotransformation and HBV replication. Its metabolites are strictly required by *A muciniphila* for functionality, because live *A muciniphila* with depleted *A muciniphila*-CM had difficulty eliminating HBV when fed to HBV-HDI mice. These metabolites are produced when *A muciniphila* consumes the nutrients from modified GAM broth. *A muciniphila* colonized in HBV-HDI mice may be inefficient in synthesizing these metabolites, which were present in high concentrations in the *A muciniphila*-CM, because of the deficiency of nutrient sources in the mouse gut.

Some bioactive metabolites within *A muciniphila*-CM were revealed in this study. One of the antiviral compounds of *A muciniphila*-CM is the nucleoside analog ribavirin, which is successful in curing patients with HCV infection because it augments the expression of antiviral type 1 cytokines.^18^ Ribavirin therapy reduces the serum levels of HBeAg, HBV-DNA, and ALT in CHB patients^33^^,^^34^; however, the outcomes are not satisfactory in treating patients with HBeAg-negative CHB.^35^ In our study, the administration of ribavirin to HBV-transfected Huh-7 cells lessened the extracellular levels of HBeAg and HBsAg but had no effect on HBV-DNA. Instead, incubation of these HBV-positive cells with *A muciniphila*-CM significantly decreased the amounts of extracellular HBeAg, HBsAg, and even the intracellular and extracellular HBV-DNA. It seems that the antiviral efficacy of *A muciniphila*-CM may be exerted through the synergistic action of its composition compounds in addition to ribavirin. Other unidentified and identified metabolites, such as apigenin, 4-methoxycinnamic acid, lovastatin, cefaclor, and benzoic acid, may add to the roles of ribavirin in *A muciniphila*-CM to enhance the antiviral effectiveness.

*R gnavus* mediated the bioconversion of CA, DCA, CDCA, α/β-MCA, and HDCA from cholesterol. A recent study demonstrated that treatment with CDCA, DCA, and α-MCA obstructs the activation and expansion of mouse primary T cells and human Jurkat T cells by abrogating the activities of store-operated calcium entry and mitochondrial calcium uptake, which are the key mechanisms for T-cell activation.^36^ Following this, a cholestasis coupled with HBV transfection mouse model, which has high levels of intrahepatic and serum BAs, revealed aggravated persistence of HBV.^36^ This finding enlightens the fact that *R gnavus–*induced biotransformation of BAs truly accounts for the more persistent aggressiveness of HBV infection.

In summary, our data provided a new perspective on the mechanism of CHB development, and the gut microbiota are involved in this process by playing dual roles in regulating HBV persistence and clearance. A key step to promote switching from the IT to IA phase is to lessen the richness of *R gnavus* and BAs bioconversion from cholesterol. The secretory products of *A muciniphila* that successfully ameliorate the burden of *R gnavus* outgrowth can be provided as useful means to induce anti-HBV efficacy. Also, the development of targeted probiotics or prebiotics that can modulate the gut microbiota composition to favor the beneficial effects of *A muciniphila* while inhibiting the detrimental effects of *R gnavus* may have translational value for CHB treatment.

## Materials and Methods

### Patient Enrollment and Sample Collection

Patients with CHB characterized by the presence of HBsAg, HBeAg, and HBV-DNA in the serum for longer than 6 months were included in this study. We excluded the CHB patients who combined with other chronic diseases and who received antibiotic therapy. A total of 102 CHB patients aged 1–58 years (M:F = 46:56) were enrolled in this study. All of them were prospectively followed up every 6 months for an average of 3.5 years, and the visit interval was shortened if liver inflammation occurred and/or if patients had received anti-HBV therapy. Patients were categorized in 3 groups (IT, IA, and HBe-SC) based on the disease courses that are categorized by liver function test and serum HBeAg positivity. IT group expressed high titer of HBeAg for more than 6 months but ALT ≤ 40 U/L. IT patients of ≥26-year age group who received FibroScan test showed absence of liver injury or had minimal steatosis or fibrosis. IA group was characterized by the presence of HBeAg and fluctuating level of ALT (>40 U/L). HBe-SC group had developed anti-HBe, absence of HBeAg in the blood, and ALT was normalized to <40 U/L.

Among 40 IT patents, 10 of them (25%, 10/40) entered the IA phase during follow-up. For IT patients aged ≥26 years (n = 12), 91.7% (11/12) had neither steatosis (controlled attenuation parameter <248 dB/m) nor cirrhosis (liver stiffness measurement <7 kPa). There is an exception that one IT patient aged 50.4 years showed controlled attenuation parameter = 268 (S1, mild steatosis) and liver stiffness measurement = 8.5 (F2 fibrosis stage). Thirty IA patients included 10 transited from IT group, and the other 20 had been already at IA phase when recruitment to this study. A total of 15 (50%, 15/30) IA patients who had ALT flares (>80 U/L) more than twice in half a year with interval >3 months received anti-HBV therapy according to the reimbursement policy of Taiwan’s National Health Insurance. HBe-SC patients were further subdivided into spontaneous HBe-SC (n = 21, 2 from IA group) and HBe-SC with antiviral therapy (n = 18, 4 from IA group). Before achieving spontaneous HBe-SC, IA patients experienced more than 2 episodes of acute exacerbation within 3 months and mean peak ALT levels = 104 U/L, of which the highest records are >300 U/L. Although the acute exacerbation of ALT in these spontaneous HBe-SC patients had not yet met the aforementioned treatment reimbursement guideline, they had successfully primed their immune systems to induce anti-HBe production and HBeAg elimination.

Blood (5∼10 mL) samples were collected at each visit and routinely monitored for the serum levels of ALT and HBV serologic markers including HBsAg, HBeAg, anti-HBs, and anti-HBe. Fecal samples (1 g) were collected from each patient for at least 5 time points; those are before and after the manifestation of liver inflammation (surrogated by flare-up of ALT >40 U/L), during and after antiviral treatment, as well as at the time of HBe-SC. This study was approved by the Institutional Review Board of the National Taiwan University Hospital Research Ethics Committee (approval numbers 201601021RINA and 201912060RINB), and informed consent was obtained from all participants aged ≥18 years and from their parents if the recruited patients were ≤18 years old.

### Generation of HBV-HDI Mouse Models With Bacterial Supplementation

Male BALB/c (3-week-old), C3H/HeN (3- and 5-week-old), and C57BL/6J (5-week-old) mice were purchased from the National Laboratory Animal Center (Taipei, Taiwan) and bred in the Animal Center of the College of Medicine, National Taiwan University. Mice were housed in ventilated caging units under positive pressure in an animal biosafety level 2 facility with standard 12-hour light-dark cycle and free access to mouse chow and sterile water. These mice were adapted for 2–7 days before performing HBV-HDI. The germ-free C3H/HeN mice were bred in the National Laboratory Animal Center, Taiwan. All the experiment procedures were approved by the Institutional Animal Care and Use Committee (IACUC) of National Taiwan University College of Medicine (IACUC approval no. 20160013 and 20190374).

The previously described plasmid pAAV/HBV1.2 (genotype A) was used for HDI.^37^ Ten μg of this plasmid was dissolved in 8% body weight of 1× phosphate-buffered saline and injected into tail veins of mice. The injection time was accomplished within 5–7 seconds. After the establishment of HBV infection, intragastric gavage of live *R gnavus* (1 × 10^6^ in 200 μL modified GAM/mouse) and *A muciniphila* (1 × 10^9^ in 200 μL modified GAM/mouse) was performed. Administration of an equivalent volume of modified GAM broth was also done in parallel to the Ctrl mice. HBV-HDI mice were fed with rodent chow (Lab Diet 5053, Purina Mills, Richmond, IN). Two other groups of HBV-HDI mice were fed with Lab Diet 5053 containing 0.5% (w/w) CA (Sigma-Aldrich, St Louis, MO) or 2% (w/w) cholestyramine (C4650, Sigma-Aldrich). Time schedules of HBV injection, sample collection, bacterial administration, and death were schematically illustrated in the respective figures.

### Analysis of HBV Serologic Markers

Detection of serum HBeAg and anti-HBe of CHB patients was accomplished using chemiluminescent microparticle immunoassays (Abbott Laboratories, North Chicago, IL). The serum levels of HBsAg, HBeAg, anti-HBs, and anti-HBe of mice were measured using the Elecsys immunoassay (Roche Diagnostics International AG, Rotkreuz, Switzerland) by an automated analyzer (Cobas e 411, Roche). All analyses were performed according to manufacturer’s protocols.

### Quantification of HBV Viral Load

Total DNAs were extracted from liver and serum samples of HBV-HDI mice and subjected to quantification of HBV copy numbers. Briefly, DNAs were extracted from 100 μL serum samples using MagNA Pure LC Total Nucleic Acid Isolation Kit (Roche, product no. 03038505001) by means of MagNA Pure LC Instruments. In addition, Puregene Core Kit A (QIAGEN Inc, Valencia, CA) was used to purify DNAs from 10 mg liver tissues. Plasmid pHBV-48 was applied to generate the standard curve of copy numbers. The standard curve was produced by serial dilutions of the plasmid ranging from 10^2^ to 10^6^ copies/mL. The PCR was performed in a total volume of 10 μL, comprising 50 ηg DNA template, 1 μL LightCycler FastStart DNA Master Hybridization Mixture (Roche), 0.8 μL of 25 mmol/L MgCl_2_, 0.3 mmol/L of each probe, and 5 mmol/L of each primer. The mixtures were processed for real-time quantitative PCR using LightCycler 480 System. The reaction was initially denaturated at 95°C for 10 minutes, followed by 45 amplification cycles of 95°C for 5 seconds, 53°C for 8 seconds, and 72°C for 14 seconds. The melting curve and quantitative analysis were conducted by LightCycler analysis software, following the manufacturer’s instructions. The sequences used forward primer 5′-CCGATCCATACTGCGGAAC-3′, reverse primer 5′-GCAGAGGTGAAGCGAAGTGCA-3′, anchor probe FLU-5′-TCTGTGCCTTCTCATCTGCCGGACC-3′-P, and sensor probe 5′-TCTTTACGCGGACTCCCC-LC-Red 640-3′.

### Fecal DNA Isolation

Stool samples were applied to DNA isolation using QIAamp PowerFecal Pro DNA kit (QIAGEN, Hilden, Germany) according to the manufacturer’s instructions. The quality and quantity of DNA were monitored by Nanophotometer and Qubit fluorometer.

### Microbial 16S rRNA PCR-based NGS

The purified fecal DNA were amplified by first round PCR using T3000 Thermocycler (Biometra, Archamps, France) with universal bacterial primers corresponding to the V3 and V4 regions of the 16S rRNA gene sequence. The sequences of forward and reverse primers are 5′-TCGTCGGCAGCGTCAGATGTGTATAAGAGACAGCCTACGGGNGGCWGCAG-3′ and 5′-GTCTCGTGGGCTCGGAGATGTGTATAAGAGACAGGACTACHVGGGTATCTAATCC-3′, respectively. PCR was achieved with program of 95°C for 3 minutes, followed by 25 cycles of 95°C for 30 seconds, 55°C for 30 seconds, and 72°C for 30 seconds and a final 5-minute elongation step at 72°C. The 16S V3 and V4 amplicons with 550 bp were yielded, and the PCR products were subsequent to PCR cleanup using AMPure XP beads (Agencourt Beckman Coulter, Beverly, MA) for purifying the 16S V3 and V4 amplicons from free primers and primer dimer species. The isolated amplicons were attached to dual indices and Illumina sequencing adapters with Nextera XT Index Kit (Illumina, San Diego, CA) in a second round PCR running the aforementioned program. Then, a secondary PCR cleanup with AMPure XP beads was performed. Last, the amplicon was subjected to microbial gene quantification using the Illumina Miseq NGS system.

The metagenomic data from NGS were analyzed by QIIME 2 next-generation microbiome bioinformatics pipeline. Divisive Amplicon Denoising Algorithm 2 (DADA2), an amplicon sequence variants–based pipeline within the QIIME 2 plugin, was applied to denoise, corrected amplicon errors and filtered out error and chimeric sequences. The other denoising based pipeline Deblur was also used for comparison. Both DADA2 and Deblur worked similarly well. The resulting 16S ASV data sets were used to assign bacterial taxonomy using Naive Bayes sklearn-based taxonomy classifier trained on EZBio cloud database. The annotation of sequences was also accomplished with NCBI, SILVA (SSU138), and GreenGenes (13_5) 16S rRNA database, especially when affiliating sequence reads to species, for confirming the accuracy of taxonomy classification. The effect size between groups was determined by LDA effect size (LEfSe) using MicrobiomeAnalyst.

### Immunohistochemistry

The paraffin-embedded mouse liver tissue sections were deparaffined, rehydrated, and unmasked using the Trilogy reagent (Cell Marque, Rocklin, CA) with microwave heating for 10 minutes. After cooling to room temperature, all tissue sections were blocked with UltraVision Hydrogen Peroxide Block (Thermo Fisher Scientific, Waltham, MA) for 10 minutes at room temperature, followed by UltraVision Protein Block (Thermo Fisher Scientific) at room temperature for another 10 minutes. Anti-HBcAg antibody (b0586, 1: 800 dilution, DAKO, UK) was applied to the tissue sections for overnight incubation at 4°C before being thoroughly washed 3 times with 1× phosphate-buffered saline. The subsequent immunostaining was carried out using an UltraVision Quanto Detection System HRP kit (Thermo Fisher Scientific) according to the manufacturer’s instructions.

### Immunofluorescence Assay

Paraffin-embedded intestinal tissue sections were deparaffined and rehydrated by immersing them in xylene and gradually diluted ethanol solutions (100%, 95%, 80%, 75%, 50%, and 0%). Tissues were then subjected to antigen retrieval using antigen retrieval buffer Tris–EDTA at pH 9.0 and heating for 20 minutes by the Bio SB TintoRetriever Pressure Cooker. After cooling to room temperature, tissue sections were treated with 3% H_2_O_2_ for 10 minutes at room temperature, washed, and then blocked for 1 hour at 37°C. The primary antibodies, ZO-1 (GTX108592, 1:100 dilution; GeneTex, Irvine, CA), were incubated with the tissue sections at 4°C overnight, and Alexa Fluor 488 Donkey anti-Rabbit IgG (H+L) Highly Cross-Adsorbed (A-21206, 1: 200 dilution; Thermo Fisher Scientific) was used as the secondary antibody. Nuclei were labeled with Hoechst 33342 (Thermo Fisher Scientific).

### Hematoxylin-Eosin Staining

The paraffin-embedded liver tissue sections were deparaffined and rehydrated to distilled water. These sections were stained with hematoxylin (Sigma-Aldrich) for 5–10 minutes, rinsed with water for 5–15 minutes, and then dehydrated by immersing through graded dilution of ethanol (50%, 75%, 80%, 95%, and 100%). After dehydration, tissue sections were stained with Eosin Y-solution 0.5% aqueous (Sigma-Aldrich) for 1 minutes, washed with 2 changes of 100% ethanol 5 minutes each, and then unmasked in 3 changes of xylene (10 minutes for each change). Finally, the tissue sections were mounted.

### Quantitative PCR

Total RNAs of mice liver tissues were isolated with the NucleoZOL Reagent (Macherey-Nagel GmbH & Co. KG, Germany). Complementary DNA was synthesized using the high-capacity cDNA Reverse transcript kit (Applied Biosystems, Foster City, CA). Real-time quantification PCR was performed in a total volume of 20 μL containing 25 ηg complementary DNA, 8 μmol/L of each primer, and 10 μL 2X SYBR Green PCR Master Mix (Applied Biosystems) on an ABI Prism 7500 Fast System (Applied Biosystems). Mouse glyceraldehyde 3-phosphate dehydrogenase was detected in parallel as an internal control. The primers used included HBcAg forward 5′-CACCGCCTCAGCTCTGTATC-3′ and reverse 5′-ACCCACCCAGGTAGCTAGAG-3′; mouse FXRα forward 5′-TGGGCTCCGAATCCTCTTAGA-3′ and reverse 5′-TGGTCCTCAAATAAGATCCTTGG-3′, as well as mouse GAPDH forward 5′-GCATGGCCTTCCGTGTTCCTAC-3′ and reverse 5′-GTCCACCACCCTGTTGCTGTAG-3′.

### Untargeted Metabolomics Analysis

For metabolites extraction, 50 mg fecal samples and liver tissues were mixed with 1 mL extract solution (acetonitrile: methanol: water = 2:2:1), and the mixtures were homogenized for 4 minutes and sonicated for 5 minutes on ice. Then the samples were incubated at –40°C for 1 hour and centrifuged at 15,000*g* for 15 minutes at 4°C. The supernatants were collected for metabolic exploration using UHPLC-Orbitrap-MS (Thermo Fisher Scientific, Bremen, Germany). Serum samples (100 μL) were mixed with 300 μL methanol, sonicated for 5 minutes in ice-water bath, incubated at –20°C for 1 hour, and centrifuged at 13,000*g* for 15 minutes at 4°C. The resulting supernatants were harvested. Quality control samples were prepared by mixing an equal aliquot of the supernatants from all samples. Ten μL of each sample was injected into a Vanquish focused UHPLC-Orbitrap Elite/MS. UHPLC separation was achieved on a 100 mm × 2.1 mm Acquity BEH 1.7 μm C18 column (Waters Corp., Milford, MA) thermostatted at 40°C, using a gradient elution profile and a binary mobile phase consisting of solvent A (deionized water containing 0.1% formic acid) and solvent B (acetonitrile with 0.1% formic acid). The flow rate was adjusted at 0.25 mL/min with a linear gradient elution over 15 minutes. Each quality control injection was done after every 5 sample injections for the peak area normalization.

Mass spectrometry data were collected in the full-scan mode of positive and negative ions with mass scan range of 70–1000 m/z. The raw data were processed with R package XCMS (version 3.2) for peak detection algorithm after converting into mzML format using ProteoWizard software. Each sample peak was normalized and quantified using area under the curve, and the metabolites were annotated using MS2 database (BiotreeDB V2.1). The cutoff for annotation was set at 0.3.

### Targeted Bile Acid Metabolomics Analysis

To purify BA metabolites from human fecal samples, 10 mg of each sample was mixed with 500 μL of MeOH/ACN/H_2_O mixture (2:2:1, v/v/v) containing 0.1% formic acid and deuterated BAs as internal standard for recovery calculation. The samples were then homogenized at 35 Hz for 4 minutes and sonicated for 5 minutes in ice-water bath, followed by –20°C incubation for 1 hour and centrifuged at 13,000*g* for 15 minutes at 4°C. The supernatant (200 μL) was collected for BA analysis. For bacterial culture supernatant samples, 100 μL of each sample was mixed with 290 μL methanol and 10 μL deuterated BAs. Samples were evenly mixed and incubated on ice for 30 minutes and, subsequent to centrifugation at 12,000*g*, at 4°C for 30 minutes. The resulting supernatants were harvested, and 200 μL of each sample was aliquoted for BA analysis using Waters ultra-high-performance liquid chromatography coupled with Waters Xevo TQ-S mass spectrometer (Waters Corp).

Chromatographic separation was accomplished on a Waters ACQUITY BEH C8 column (2.1 mm × 100 mm, particle size 1.7 μm; Waters Corp). Column temperature was maintained at 60°C with mobile phase A (10% acetonitrile and 0.01% formic acid) and mobile phase B (isopropanol/acetonitrile [50:50, v/v] and 0.01% formic acid). Mass spectrometer was operated in positive-ion ESI mode. The capillary voltage was set at 1.5 KV. Desolvation gas flow rate was 1000 L/h, and cone gas flow was maintained at 150 L/h. The desolvation and source temperatures were set at 600°C and 150°C, respectively. Quality control samples and pooled quality control samples (a mixture of all samples) were analyzed after every 10 samples, and the quantitative results were compared to assess the quality of each assay. Data were calculated using Waters TargetLynx Application Manager.

### Expression and Activity Detection of BSH

Full-length *BSH* gene encoded by *R gnavus* genome was obtained by gene synthesis technique and cloned into the pET-28a(+) plasmid (Sigma-Aldrich) by NdeI and XhoI digestion. Plasmid pET-28a(+)-BSH was transformed into *Escherichia coli BL21* (DE3; Thermo Fisher Scientific) to express BSH protein, which was then purified, and the molecular weight was confirmed by Coomassie blue (Thermo Fisher Scientific)-stained sodium dodecyl sulfate–polyacrylamide gel electrophoresis. The purified BSH protein was subjected to the following BSH activity analysis.

Primary BAs, including TCA, GCA, TCDCA, T-α-MCA and T-β-MCA, were prepared as substrates. A total 200 μL reaction composed of 178 μL sodium-phosphate buffer (0.1 mol/L, pH 6.0), 10 μL purified BSH, 10 μL of 100 mmol/L primary BAs, and 2 μL of 1 mol/L DTT was mixed and incubated at 37°C for 30 minutes. Then, 50 μL of this reaction mixture was aliquoted into a new tube, mixed with 50 μL of 15% (w/v) trichloroacetic acid, and centrifuged at 13,000*g* for 10 minutes. The resulting supernatant contained the glycine or taurine that deconjugated from the primary BAs. Then, 50 μL of this supernatant was incubated with 950 μL of ninhydrin reagent (1% [w/v] ninhydrin in 0.5 mol/L sodium-citrate) at 100°C water bath for 15 minutes, followed by cooling in ice. The concentration of the deconjugated glycine or taurine, which represents the BSH activity, was determined by measuring the absorbance at 570 nm and estimated using standard curve plotted by increasing doses of glycine or taurine (0, 0.5, 1, 1.5, and 2 mmol/L). Finally, the BSH activity was calculated and expressed as μmol of amino acids released per minute per milligram of BSH.

### Antibacterial Activity Analysis

LIVE/DEAD BacLight Bacterial Viability and Counting Kit (L34856R; Molecular Probes, Inc, Eugene, OR) was adopted in this assay. Both *R gnavus* and *A muciniphila* were anaerobically cultured in modified GAM broth at 37°C. For each reaction, *R gnavus* cells (1 × 10^8^) were incubated with the green fluorescent SYTO 9 nucleic acid stain (20 μmol/L) at 37°C for 2 hours. After washing (centrifuged at 15,000*g* for 10 minutes) twice at room temperature, the bacterial pellet was resuspended in 100 μL modified GAM. *R gnavus* was then anaerobically co-cultured with an increasing amount (1 × 10^7^ to 1 × 10^10^) of *A muciniphila* or the respective CM in a total volume of 1 mL modified GAM broth for overnight at 37°C. Once washed twice with 1 mL 150 mmol/L NaCl, the bacteria were stained with 40 μmol/L red fluorescent propidium iodide in 100 μL of 150 mmol/L NaCl. The live and dead bacteria were quantitated with flow cytometer using the FITC-channel for *R gnavus* and the V450 channel for dead cell detection.

### Antiviral Activity Analysis

Huh-7 cells were grown in high glucose Dulbecco modified Eagle medium supplied with 10% fetal bovine serum, 2 mmol/L L-glutamate (Gibco, Grand Island, NY), 1 mmol/L sodium pyruvate (Gibco), 1× MEM non-essential amino acids (Gibco), and antibiotics (MicroZap Plus-CL, Lonza, Basel, Switzerland). After trypsinization, 4 × 10^5^ cells were seeded onto each well of 6-well plate and then allowed to attach overnight. Subsequently, cells were transfected with pAAV-HBV 1.2 plasmid (2.5 μg/well) using Lipofectamine 3000 (Life Technologies, Carlsbad, CA). Four hours after transfection, the culture media were refreshed, and cells were incubated with a series dosage (5, 50, 250, and 500 μL) of *A muciniphila*-CM, which was collected from live culture of *A muciniphila* (1 × 10^10^/mL) in modified GAM. Ribavirin (800 μmol/L/well, Sigma-Aldrich) was also administrated to the HBV-transfected Huh-7 cells. All these cells were cultured for another 24 hours before harvest. Culture supernatants were collected for the measurements of HBsAg, HBeAg, and HBV-DNA after centrifugation at 500*g* for 5 minutes to remove cell debris. In addition, cells lysates were harvested for assessing viral load using lysis buffer provided in Puregene Core Kit A (QIAGEN).
